# Grading fluorescein angiograms in malarial retinopathy

**DOI:** 10.1186/s12936-015-0897-7

**Published:** 2015-09-24

**Authors:** Ian J. C. MacCormick, Richard J. Maude, Nicholas A. V. Beare, Shyamanga Borooah, Simon Glover, David Parry, Sophie Leach, Malcolm E. Molyneux, Baljean Dhillon, Susan Lewallen, Simon P. Harding

**Affiliations:** Department of Eye and Vision Science, University of Liverpool, Liverpool, UK; Malawi-Liverpool-Wellcome Trust Clinical Research Programme, Blantyre, Malawi; Centre for Clinical Brain Sciences, University of Edinburgh, Edinburgh, UK; Mahidol-Oxford Tropical Medicine Research Unit, Faculty of Tropical Medicine, Mahidol University, Bangkok, Thailand; Centre for Tropical Medicine and Global Health, Nuffield Department of Medicine, University of Oxford, Oxford, UK; College of Medicine and Veterinary Medicine, University of Edinburgh, Edinburgh, UK; St. Paul’s Eye Unit, Royal Liverpool University Hospital, Liverpool, UK; Department of Ophthalmology, University of Edinburgh, Edinburgh, UK; Princess Alexandra Eye Pavilion, Edinburgh, UK; School of Medicine, University of St Andrews, St Andrews, UK; Liverpool Ophthalmic Reading Centre, St. Paul’s Eye Unit, Royal Liverpool University Hospital, Liverpool, UK; Liverpool School of Tropical Medicine, Liverpool, UK; Kilimanjaro Centre for Community Ophthalmology, Cape Town, South Africa

**Keywords:** Severe malaria, Malarial retinopathy, Fluorescein angiography, Grading, Inter-grader agreement

## Abstract

**Background:**

Malarial retinopathy is an important finding in *Plasmodium falciparum* cerebral malaria, since it strengthens diagnostic accuracy, predicts clinical outcome and appears to parallel cerebral disease processes. Several angiographic features of malarial retinopathy have been described, but observations in different populations can only be reliably compared if consistent methodology is used to capture and grade retinal images. Currently no grading scheme exists for fluorescein angiographic features of malarial retinopathy.

**Methods:**

A grading scheme for fluorescein angiographic images was devised based on consensus opinion of clinicians and researchers experienced in malarial retinopathy in children and adults. Dual grading were performed with adjudication of admission fluorescein images from a large cohort of children with cerebral malaria.

**Results:**

A grading scheme is described and standard images are provided to facilitate future grading studies. Inter-grader agreement was >70 % for most variables. Intravascular filling defects are difficult to grade and tended to have lower inter-grader agreement (>57 %) compared to other features.

**Conclusions:**

This grading scheme provides a consistent way to describe retinal vascular damage in paediatric cerebral malaria, and can facilitate comparisons of angiographic features of malarial retinopathy between different patient groups, and analysis against clinical outcomes. Inter-grader agreement is reasonable for the majority of angiographic signs. Dual grading with expert adjudication should be used to maximize accuracy.

**Electronic supplementary material:**

The online version of this article (doi:10.1186/s12936-015-0897-7) contains supplementary material, which is available to authorized users.

## Background

### Malarial retinopathy in severe malaria

The clinical syndrome of cerebral malaria (CM) is a major cause of death and disability, yet the pathogenesis remains unclear [1]. Improvements in diagnosis, treatment, and prognosis are likely to be possible only through an improved understanding of the disease process. The neurovasculature of the retina has attracted interest as a potential model of unseen cerebrovascular damage, both in CM [2] and other neurological conditions, including stroke [3], cerebral small vessel disease [4], and others [5, 6].

A system to classify and grade malarial retinopathy from ophthalmoscopy [7, 8] and colour photographs has been widely used, in both children [9, 10] and adults [11, 12]. This has led to an awareness that malarial retinopathy in paediatric CM is a sensitive and specific indicator of cerebral sequestration [13], and that the severity of retinopathy correlates with the severity of retinal and cerebral sequestration [14], the intensity of cerebral haemorrhages [15], and the likelihood of a fatal outcome [10]. Retinopathy in adults with CM appears to involve fewer features than paediatric cases, but is also associated with death and other clinical markers of disease severity [11, 12].

Fundus fluorescein angiograms (FA) have been performed on children [16, 17] and adults with CM [18]. This procedure involves injection of a fluorescent solution (fluorescein sodium) into a peripheral vein, and then taking photographs as it moves through the retinal circulation. When excited by short wavelength (blue) light fluorescein emits light of a longer wavelength (green). The light stimulus and corresponding signal are differentiated by a combination of filters, and the result is a map of retinal vessel structure and function. Structure because vessels, including capillaries, are highlighted by the dye, and function because any obstruction in or leakage from vessels is clearly seen [19, 20]. FA in paediatric and adult CM reveals several distinctive features in addition to those previously recognized from ophthalmoscopic examination or colour images of the retina [17]. Angiographic features may have important associations with cerebral pathology and clinical outcome. If such associations exist, they can only be quantified, replicated and compared between studies if observers of different populations use a consistent grading scheme. A scheme for grading FA signs in severe malaria has not yet been described. We therefore developed a grading scheme for FA images in severe malaria and tested its performance on a set of images from children with CM.

The purpose of this paper is not to propose a new diagnostic or prognostic test. Instead it is to provide the means to reliably analyse retinal images taken in the course of clinical research. Such research may eventually provide insights into cerebral malaria pathogenesis which may, in turn, ultimately inform the design of new treatments. Although we used a table-mounted camera within a specialist research ward, portable retinal cameras now provide the means to take similar retinal images on a wider range of patients [20].

## Methods

Angiographic features of CM in Malawian children and Asian adults were reviewed by an expert group with knowledge of severe malaria and malarial retinopathy, with the aim of developing terminology, definitions and selecting standard images. The grading scheme was developed from, and tested on, admission images from children admitted to the Paediatric Research Ward in Blantyre, Malawi, between 2006 and 2014. The great majority of subjects had CM; a few had other malarial or non-malarial diagnoses. A Topcon 50-EX optical unit (Topcon, Japan) was used, matched to a Nikon E1-H digital camera and desktop PC running Imagenet 2000 (Topcon, Japan) to capture both colour and FA images. FA images were taken after injection of 1–5 ml of sodium fluorescein 10–20 % into a peripheral vein as described previously [17]. Images were generally low compression JPEG, or TIFF files with a 50º field of view. Some 20º images were also reviewed. A minority of JPEG files were of lower resolution.

**Fig. 1:**
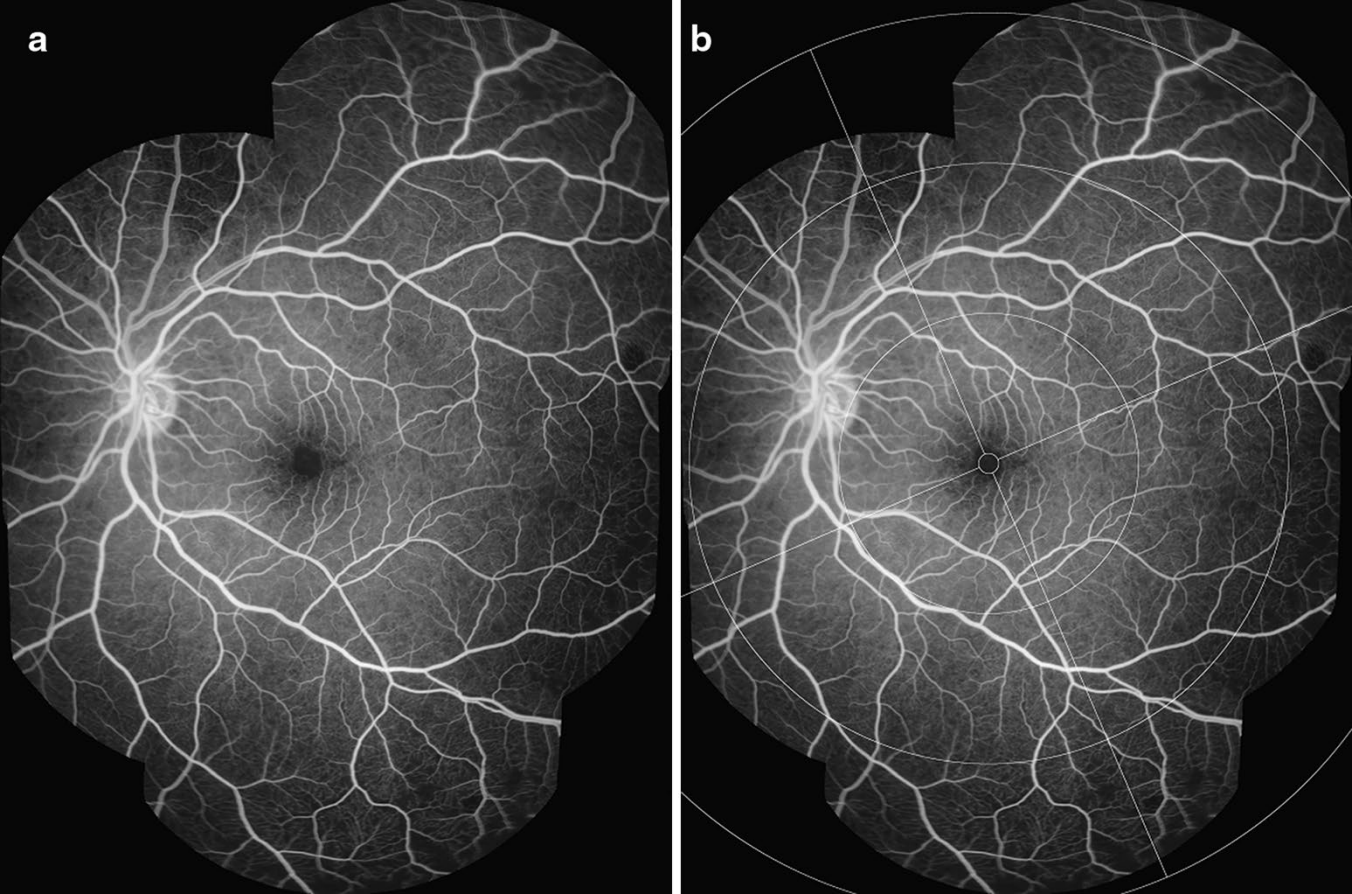
FA montage and grading overlay. **a** A montage composed of several FA images from similar time points can provide a useful summary of FA features. **b** Addition of a grading overlay assists identification of retinal areas. Detail of retinal areas are in Table 1 and Fig. 2

**Fig. 2:**
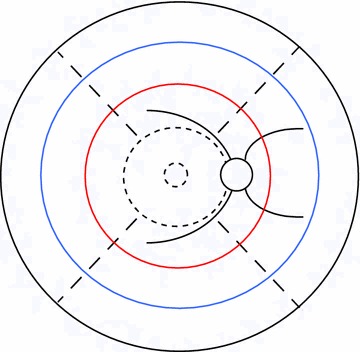
Definitions of retinal areas. Schematic retina from [8] adapted to show three different extents of peripheral retina for the *right eye* (Table 1). The disc and vessels are shown for reference. Note that the disc is always on the nasal edge of the macula. The distance from the outer edge of the disc to the centre of the fovea is referred to as one disc-fovea distance, and is equivalent to ~2.5 ETDRS disc diameters. Working from the centre of the image outwards, the fovea and macula are shown as circles (*broken lines*). The *red circle* indicates the limit of zone 1, which extends one disc-fovea distance beyond the macula. The *blue circle* indicates the limit of zone 2, which extends a further disc-fovea distance from zone 1. Zone 3 includes all retina outwith the *blue circle*. The *outer black circle* nominally represents the ora serrata, but note the actual distance between the zone 2/3 boundary and the ora is much greater than suggested by this diagram. Superior, temporal, inferior, and nasal quadrants are shown by *broken lines* extending from the edge of the macula

A grading scheme was devised (Additional file 1) and used in dual grading with independent adjudication. Grading was performed by two professional graders in the Liverpool Ophthalmic Reading Centre, St Paul’s Eye Unit, Liverpool, UK with 9.5 and 1.5 years experience in grading FA images for other retinal diseases, such as age-related macular degeneration. Images were viewed using Microsoft Office 2010 Picture Manager, on Dell P2412H screens with 1920 × 1020 resolution, and enhanced with standard tools as necessary (e.g. brightness, contrast). Montages were created using Imagenet IBase (Topcon, Japan), and a grid indicating retinal zones and quadrants was overlaid to facilitate grading and calibration (Figs. 1, 2) (In-house software, Matlab, Mathworks). Graders reviewed montages and original FA images, and could check corresponding colour images.

Disagreement between graders was defined as ≥two-level discrepancy for ordinal variables (i.e. variables with three or more levels), and any discrepancy in binary variables. Disagreement was adjudicated by one of two ophthalmologists with several years experience of FA in paediatric CM (IJCM, SPH). All observers were masked to subject identity and clinical characteristics, including outcome.

Grading data were imported to Stata 13 (StataCorp, Texas), and observed agreement was calculated for comparisons between grader 1 and grader 2. Large focal leak was compared after converting it from a count variable to an ordinal variable (no leak, one site of leak, >1 site of leak). Only data from the left eye were used.

Admission examination was performed as described previously [21]. Respiratory distress was defined as any one of alar flare, chest recession, accessory muscle use, or deep breathing [22]. Malarial retinopathy was diagnosed by an ophthalmologist on the basis of bilateral dilated indirect ophthalmoscopy and defined as any one of retinal haemorrhage, retinal whitening, or orange or white retinal vessels. Isolated papilloedema did not indicate malarial retinopathy [13]. Admission investigations were performed: peripheral parasitaemia (microscopy of a finger prick blood sample); full blood count (Coulter Counter, Becton–Dickinson, Franklin Lakes, NJ); haematocrit (finger prick sample and centrifuged microhaematocrit tube); venous lactate [Lactate Pro point of care detector (Arkray Inc)], plasma HRP2 (Celabs ELISA); HIV status (Uni-Gold—Trinity Biotech, Carlsbad, CA; and Determine—Inverness Medical, Orlando, FL).

This research was performed in accordance with the Declaration of Helsinki and was approved by the research ethics committees at Michigan State University, the University of Liverpool, and the University of Malawi College of Medicine. Informed consent to participate in the study was obtained from the parents or guardians of all participants.

## Results

Results are divided into three sections: principles of grading FA images, a description of specific FA features, and performance of the grading scheme in terms of inter-grader agreement. The grading form is provided as Additional file 1.

### Principles of grading FA images

In addition to recognizing specific retinal features, consistent grading requires attention to several concepts related to image acquisition, processing, viewing, and interpretation.

#### Image acquisition

The grading scheme used is based on observations constrained by the limits of a 50° field of view and the limits of digital resolution for TIFF or high quality JPEG files. Suboptimal image resolution interferes with grading. The authors recommend that researchers acquire wide field images in a lossless format.

#### Image processing: montages and grading overlay

Combining individual images into a montage allows a grader to view a large area of retina in one image (Fig. 1). A montage can give a helpful overview of the retina before the full image set is examined, and can be used to prevent double counting of some retinal features from individual images.

However, inevitably FA montages are made up of images that are taken at slightly different times during the fluorescein run, and the process of selecting and then combining images risks loss of information. Ideally a montage should be made up of images from similar time points, and graders should use their judgement to make a grading based on both montages and individual images.

A grading overlay is applied to montages to help determine retinal areas (Fig. 1b). A time stamp should be added to each FA image to show the time from fluorescein injection.

#### Viewing images

Images should be viewed in a darkened room on a large, high-resolution monitor (e.g. 1920 × 1200 pixel resolution). When available, information from colour images and montages of FA images should be used to help determine a grading. A good image-viewing software package with the ability to enhance the images should be used. In a formal reference grading centre a calibration is performed to correct for variable image size. Standard scale bars and discs are used as guides. Adjustments to brightness and contrast should be small in order not to lose the detailed features of lesions that were present in the original images.

#### Image interpretation

##### Retinal areas and minimum area visible

The retina is divided into several areas. The macula lies within a circle centred on the foveal centre and extending to the temporal border of the optic disc. The Early Treatment Diabetic Retinopathy Study (ETDRS) standard distance from the foveal centre to the temporal border of the disc (2.5 disc diameters, equivalent to 3.8 mm) was used to define the radius of the macula, and as a standard measure: the “disc-fovea distance” [23]. All retina beyond the macula is defined as peripheral retina. The periphery is divided into four quadrants. Each quadrant is divided into three zones, which cover areas of peripheral retina at increasing distances from the macula (Table 1; Figs. 1b, 2).

**Table 1:** Definition of retinal areas

Retinal area	Definition
Macula	A circle centred on the centre of the fovea with a radius of 2.5 ETDRS disc diameters, or 3.8 mm [23]
	The nasal edge is approximately in contact with the temporal border of the optic disc
Fovea	A circle centred on the centre of the fovea with a diameter of 1/3 disc diameter, or ~0.5 mm
Zone 1 (inner periphery)	The area between the macula and a circle extending 1 disc-fovea distance from the edge of the macula (Fig. 1, red-circle)
Zone 2 (mid periphery)	The area between the circle defining the extent of the inner periphery and a circle extending two disc-fovea distances from the edge of the macula (Fig. 2, blue circle)
Zone 3 (far periphery)	All retina beyond the extent of the mid periphery (Fig. 2, beyond the blue circle)

Retinal areas are defined in terms of quadrants centred on the fovea, and concentric rings at increasing distance from the fovea. Distances are given in terms of retinal landmarks, such as proportions of optic disc diameter, and the distance from the disc to the fovea

Each retinal area is deemed visible for the purposes of grading if ≥75 % of the area is captured in the image sequence. The exception is zone 3 which is deemed visible for a given quadrant if any retina is visible beyond the limit of zone 2. The amount of retina visible for grading is recorded on the grading form (Additional file 1).

##### Types of vessel segment

Some FA features in CM are localized to particular segments of vessels, and particularly venules. The simplest method to classify microvessels is to divide vessels into idealized groups. Other approaches include Horton–Strahler ordering and generation numbering [24]. Large retinal vessels often have multiple small 90° branches in between dichotomous branches. It is difficult for a human grader to describe this arrangement according to generation number or Horton–Strahler order. Vessels are grouped according to several types (Table 2).

**Table 2:** Definition of vessel types

Vessel type	Description
Capillary	Smallest vessel visible on a well focussed angiogram image
Post-capillary venule	Formed by the confluence of two or more capillaries, and extends up to the point where it is joined by a second post-capillary venule or other larger venular segment
Pre-capillary arteriole	Extends upstream from the divergence of two or more capillaries to the point where it branches from another pre-capillary arteriole or other larger arteriolar segment
Post-capillary venule complex	Extends approximately 1/3 of a disc diameter (~500 μm) downstream from the capillaries along venular segments
Pre-capillary arteriole complex	Extends approximately 1/3 of a disc diameter (~500 μm) upstream from the capillaries along arteriolar segments
Small venule	Extends from the edge of the post-capillary venule complex downstream to the point of confluence with another venule of similar or larger caliber
Small arteriole	Extends from the edge of the pre-capillary arteriole complex upstream to the point of branching with another arteriole of similar or larger caliber
Large venule	Extends downstream from the point of confluence that defines the upper caliber boundary of small vessel segments to the edge of the optic disc
Large arteriole	Extends upstream from the branching point that defines the upper caliber boundary of small arteriolar segments to the edge of the optic disc

##### Definition of vessel types: capillaries and the post-capillary venule complex

Capillaries are the smallest vessels visible on a well-focussed angiogram. A post-capillary venule is formed by the confluence of two or more capillaries, and extends up to the point where it is joined by a second post-capillary venule or other larger venular segment.

**Fig. 3:**
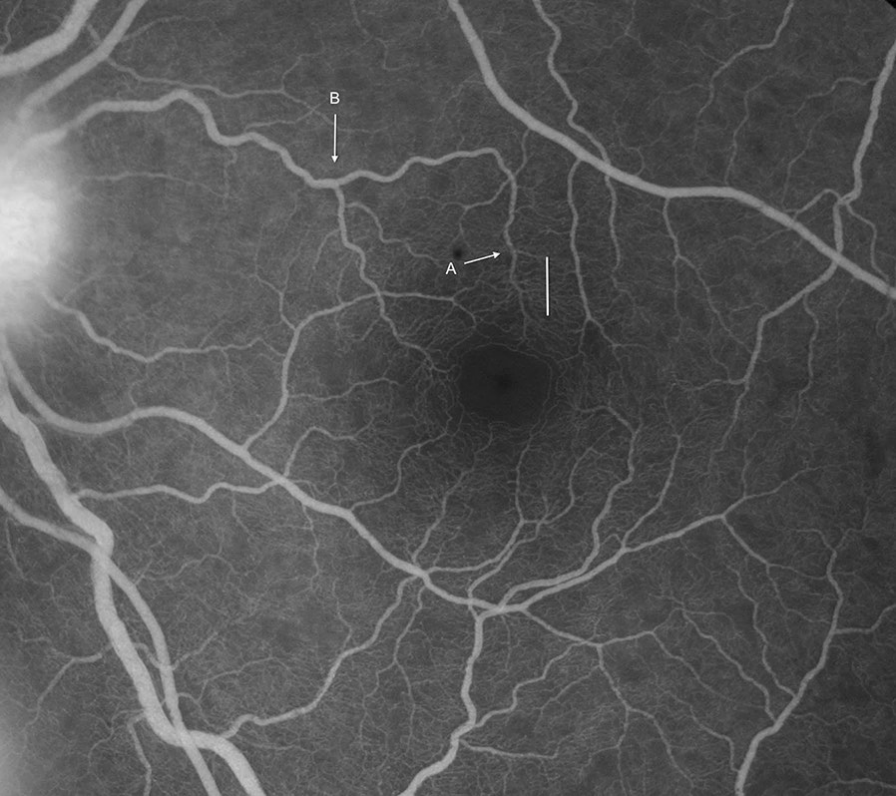
The post-capillary venule complex, small venules, large venules. *Left eye* *point A* represents the beginning of a ‘small venule’ segment, which ends at *point B* when two small venules converge. *Point B* is also the beginning of a large venule, which ends at the optic disc. The *scale bar* shows 1/3 disc diameter (~500 μm) from the convergence of capillaries on to the post-capillary venule, and approximates the length of the post-capillary venule complex. The post-capillary venule complex begins at the junction of two capillaries and extends a distance of 1/3 disc diameter downstream towards the small venules. Other visible features in this figure include disc leak, and intravascular filling defects (e.g. small venule at *point A*)

It is more practical to identify a larger group of vessels (the post-capillary venule complex), which extend approximately 1/3 of a disc diameter (~500 μm) downstream from capillaries (Fig. 3). An analogous selection of pre-capillary arterioles can also be considered (the pre-capillary arteriole complex), which includes arteriolar segments 1/3 of a disc diameter upstream from the capillary bed.

##### Definition of vessel types: small and large venules

**Fig. 4:**
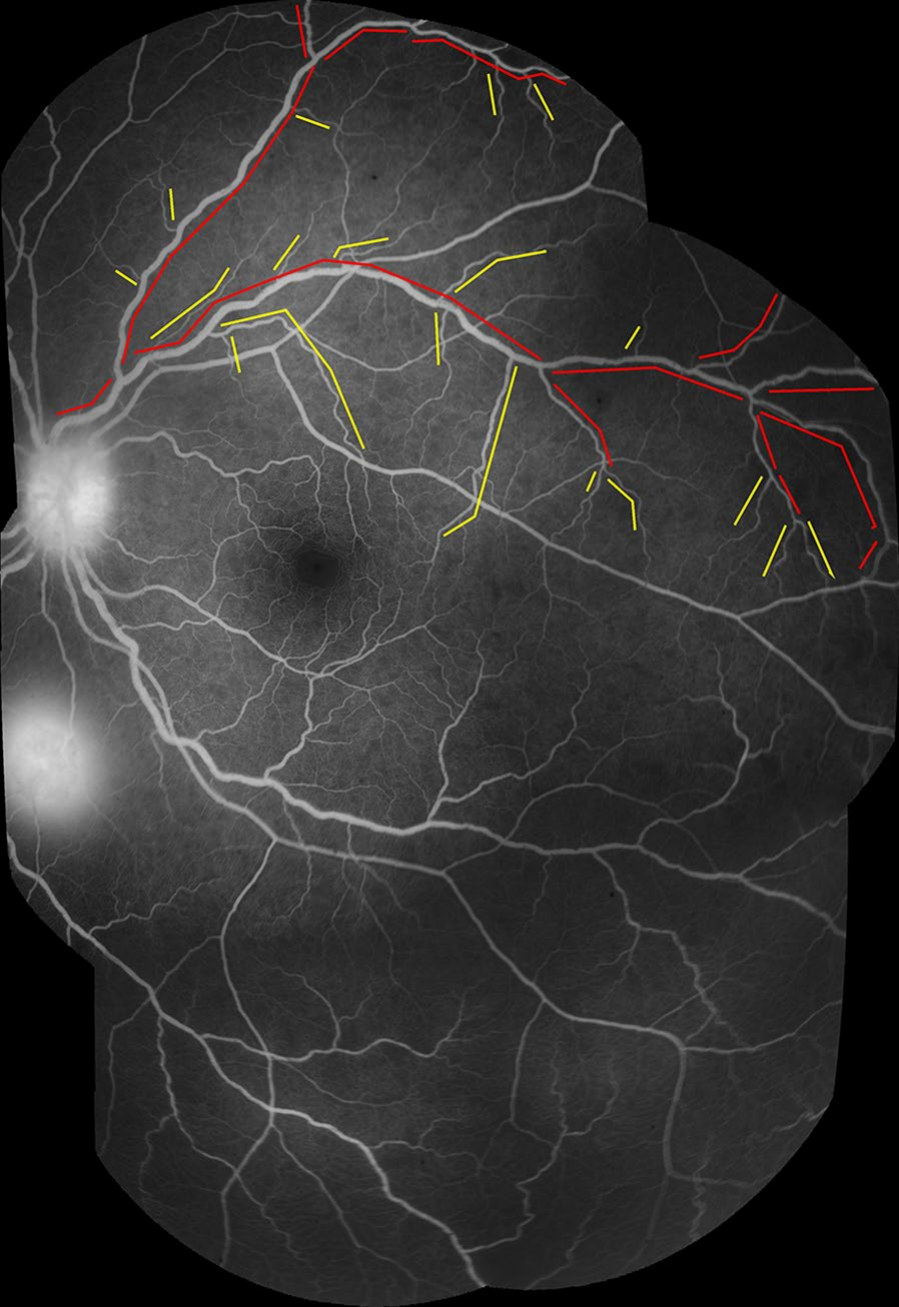
Segments of the venous network (small and large venules). Montage of FA images (*left eye*). Large vessel segments are marked in *red*, and small vessel segments in *yellow*. A *break in a line* indicates the junction between two vessel segments. New vessel segments begin where a vessel meets another vessel of similar or larger calibre. Small vessels extend from the downstream limit of the post-capillary venule complex (~500 μm from capillary bed) to the point where the small vessel joins another vessel of similar or larger calibre. Large vessels extend from this point to the disc. Sections of large vessels begin and end at junctions between one large vessel and another large vessel of similar or greater calibre. Other visible features in this figure include disc leak, and one site of large focal leak (inferior to the disc)

Small venules are defined as any section of vein between the edge of the post-capillary venule complex up to the point of confluence with another vessel of similar or larger calibre. Large venules extend from the point where two small venules converge to the edge of the optic disc (Fig. 3). Separate sections of large vessels can be counted, since they begin and end at junctions between one large vessel and another large vessel of similar or larger calibre (Fig. 4).

Analogous definitions exist for retinal arteriole segments (Table 2). Arterioles fill early in the angiogram and are generally narrower than venules, which fill later.

##### Grading image quality

Patients with CM are acutely unwell. They may be restless, and may have tonic or roving eye movements, nystagmus, or reduced corneal transparency secondary to incomplete lid closure. Grading of one or more features may be impossible if images are blurred, features are obscured by haemorrhages or leaking fluorescein, or if the area of retina to be graded has not been captured at an appropriate time point. Blurred images are a particular problem for grading vessel leak and intravascular filling defects (IVFD) (see sections on specific lesions).

Overall image quality is graded by evaluating the sharpness of FA images (Table 3). A score for image quality is assigned to the whole run for FA images for a given eye (Additional file 1). Grades for individual retinal features should only be assigned when the grader is ≥50 % certain that a feature is present, absent, or present at a given level of severity. A grade of ‘cannot grade’ (CG) is given if the grader is unable to tell if a feature is absent, present, or how severe it is on an ordinal scale. CG is not the same as ‘absent’. ‘Absent’ means the grader is ≥50 % certain the feature is not present. The purpose of the CG category is to prevent an accumulation of false negative data by ensuring that subjects are not graded as having a lack of retinal signs when in fact they just have poor quality images.

**Table 3:** Definition of overall image quality for all images from a given eye

Image quality	Definition
Good	Focus and clarity are sufficient for grading of all features to be completed
	Retinal details (small capillaries) are sharply defined and have crisp boundaries. It should be possible to see the ends of the larger vessels approaching the foveal avascular zone (FAZ), if they are not obscured by pathology
Fair	The image is less well focused (than good), with it becoming more difficult to determine the ends of the larger vessels approaching the FAZ
	Retinal details are slightly fuzzy but subtle lesions, such as small retinal haemorrhages, can still be seen and graded
Poor gradeable	The image is less well focussed (than fair), but it is possible to glean some information for grading
	Clarity is decreased so that subtle lesions might be missed, but is sufficient for assessment of large retinal haemorrhages and large retinal vessels
Poor ungradeable	This should be selected if the grader is unable to evaluate or distinguish (with more than 50 % confidence) the absence or presence of any feature in all of the available images

## Specific FA features in malarial retinopathy

### Macular capillary non-perfusion (CNP)

CNP is an area of the capillary network that fails to fill with fluorescein by the late arteriovenous phase, with minimum linear dimension (MLD) ≥63 μm. 63 μm is approximately ½ the width of a retinal venule at the disc margin and is a conventional size circle used in the grading of drusen for age-related macular degeneration.

**Fig. 5:**
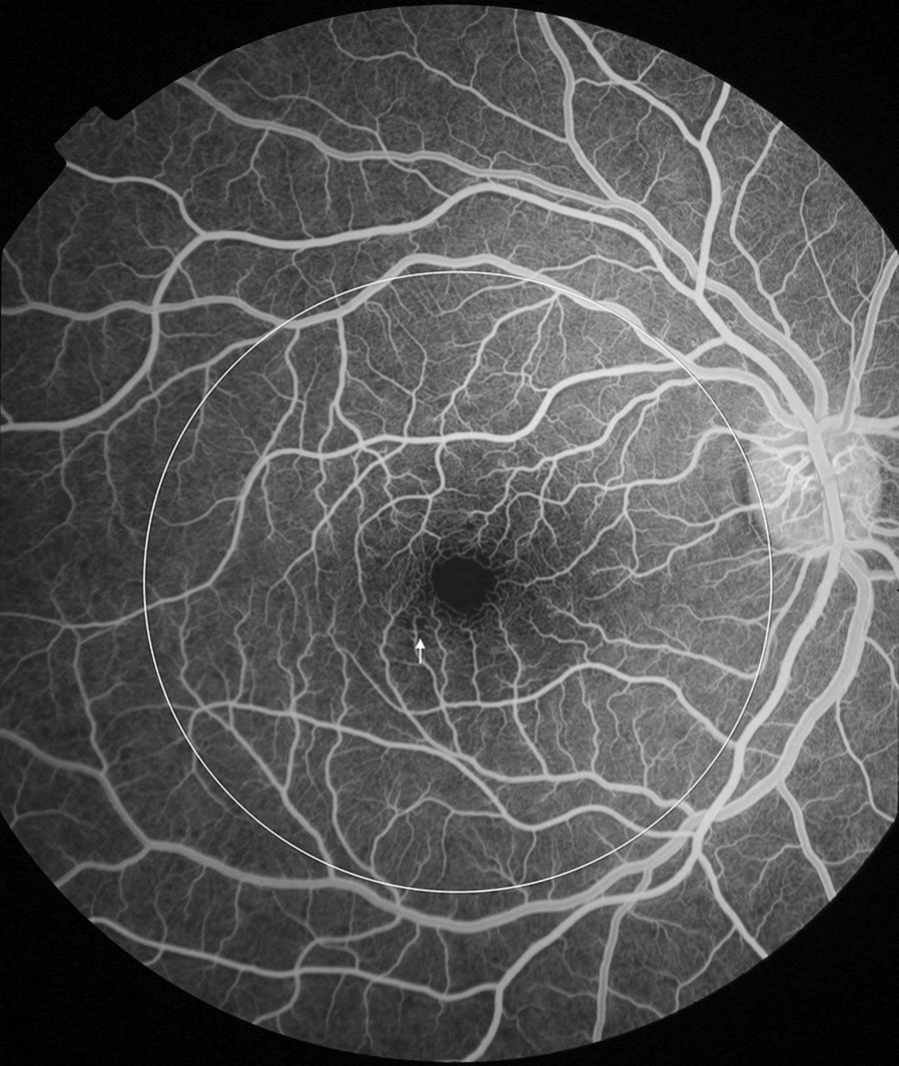
Grade 1 macular capillary non-perfusion (CNP). *Right eye* a few small areas of CNP are seen at the macula (*arrow*), which is outlined by a *white circle*

**Fig. 6:**
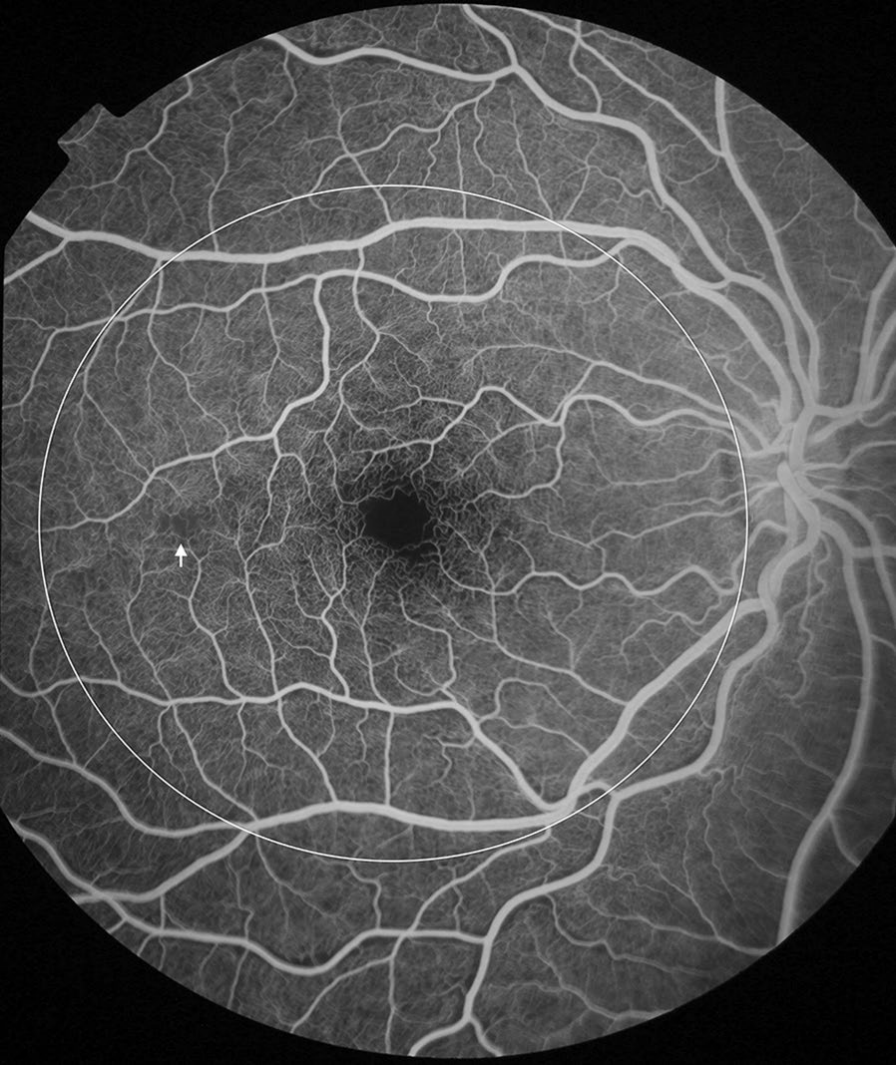
Grade 1 macular capillary non-perfusion (CNP). *Right eye* a few small areas of CNP are seen at the macula (*arrow*), which is outlined by a *white circle*

**Fig. 7:**
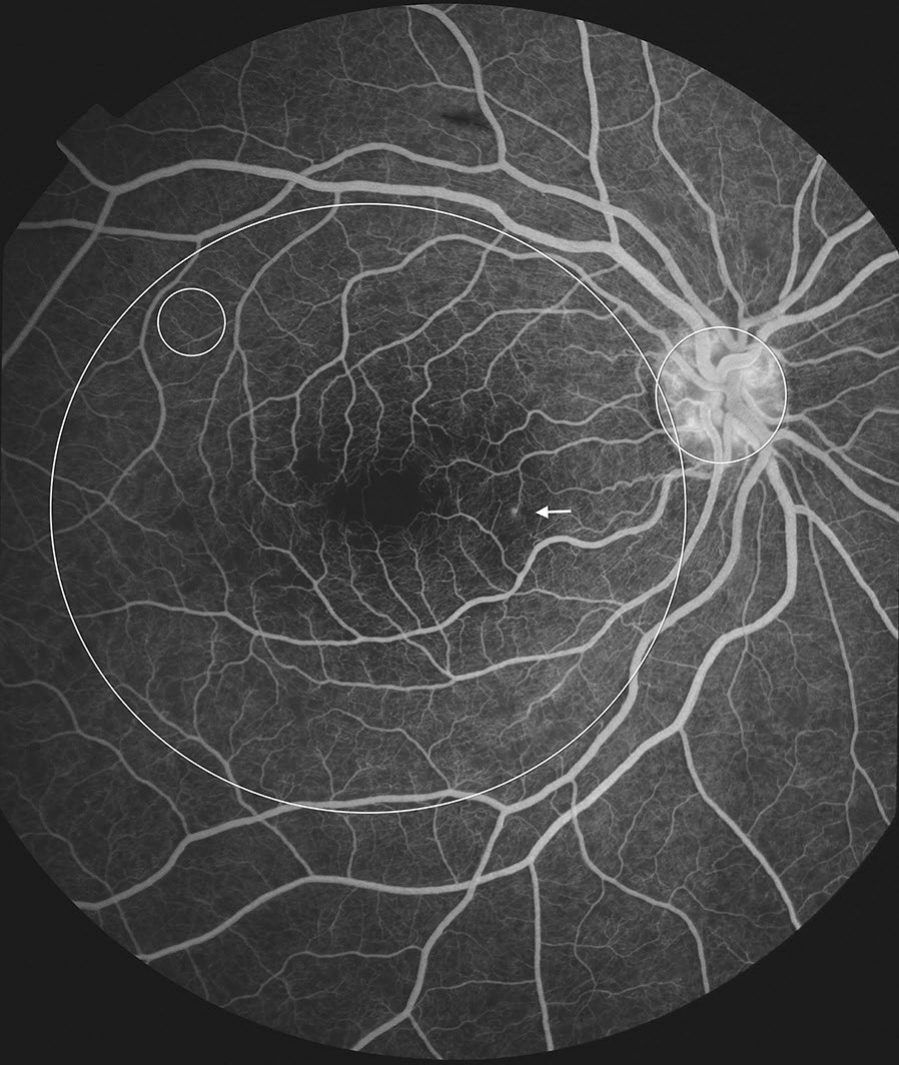
Grade 2 macular capillary non-perfusion (CNP). *Right eye* the upper limit of grade 2 macular CNP. Combined area of macular CNP is <1/3 disc area, after mentally subtracting a notional area for the normal foveal avascular zone. Macula, disc, and 1/3 disc area are shown as *white circles*. A single site of punctate focal leak is also visible in the macula (*arrow*)

**Fig. 8:**
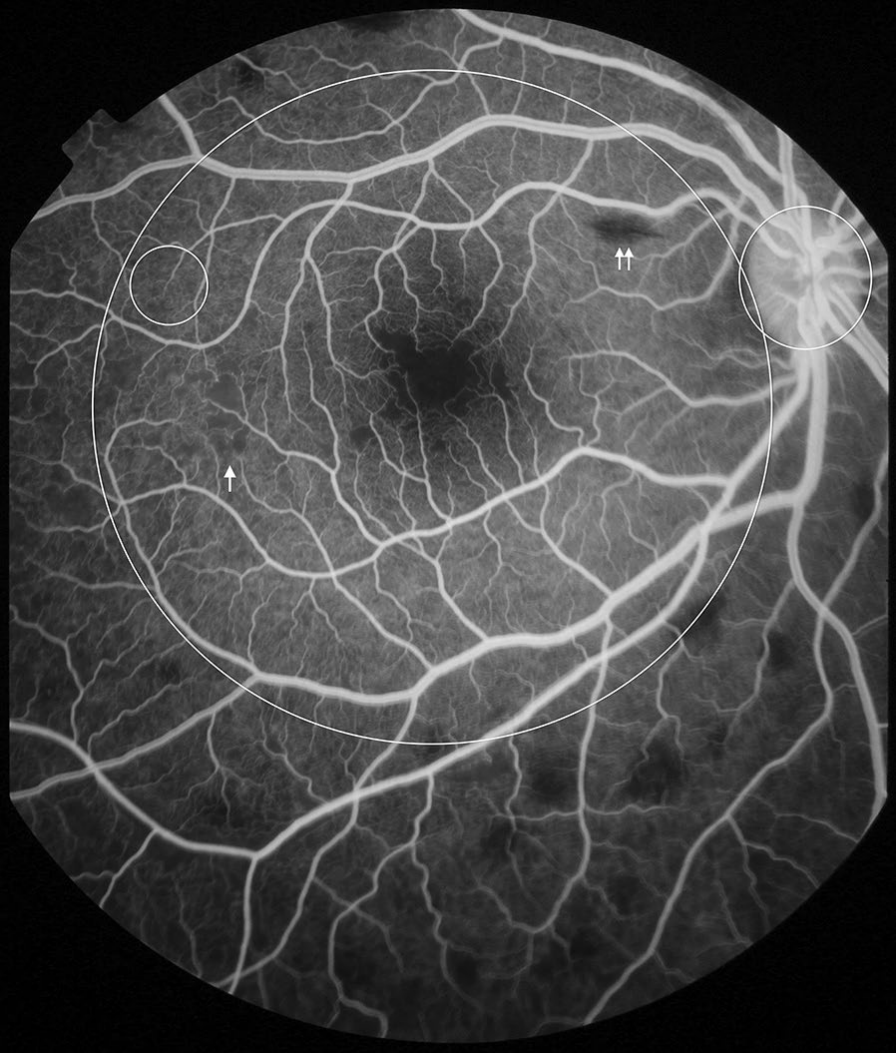
Grade 2 macular capillary non-perfusion (CNP). *Right eye* the upper limit of grade 2 macular CNP. Combined area of macular CNP is <1/3 disc area. CNP is clearly seen around the foveal avascular zone, and less obviously in the temporal macula (*arrow*). Macula and 1/3 disc area are shown as *white circles*. Masking of fluorescein from haemorrhage is also visible (*double arrow*). CNP has geographic boundaries (e.g. the abnormally irregular edge of the foveal avascular zone in this image), while haemorrhages tend to have rounded edges

**Fig. 9:**
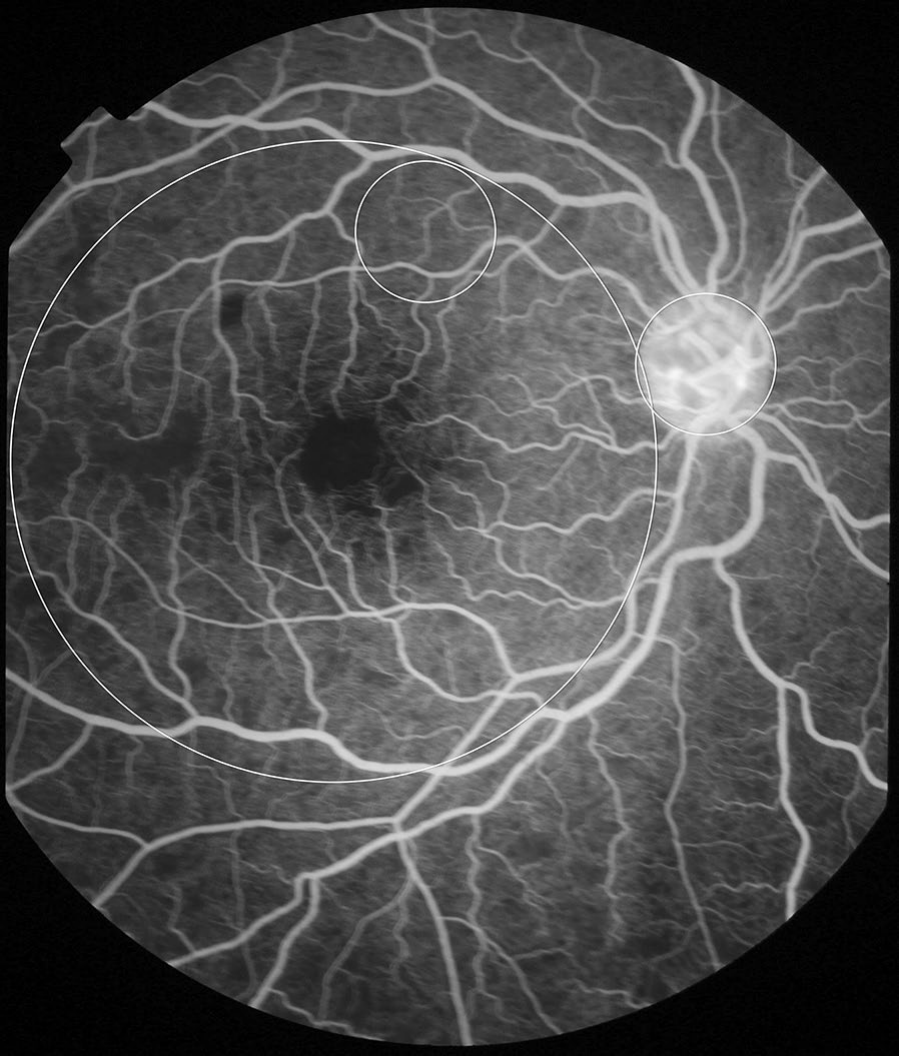
Grade 3 macular capillary non-perfusion (CNP). *Right eye* the upper limit of grade 3 macular CNP. Combined area of macular CNP is 1/3–1 disc area. Macula and one disc area are shown as *white circles*

**Fig. 10:**
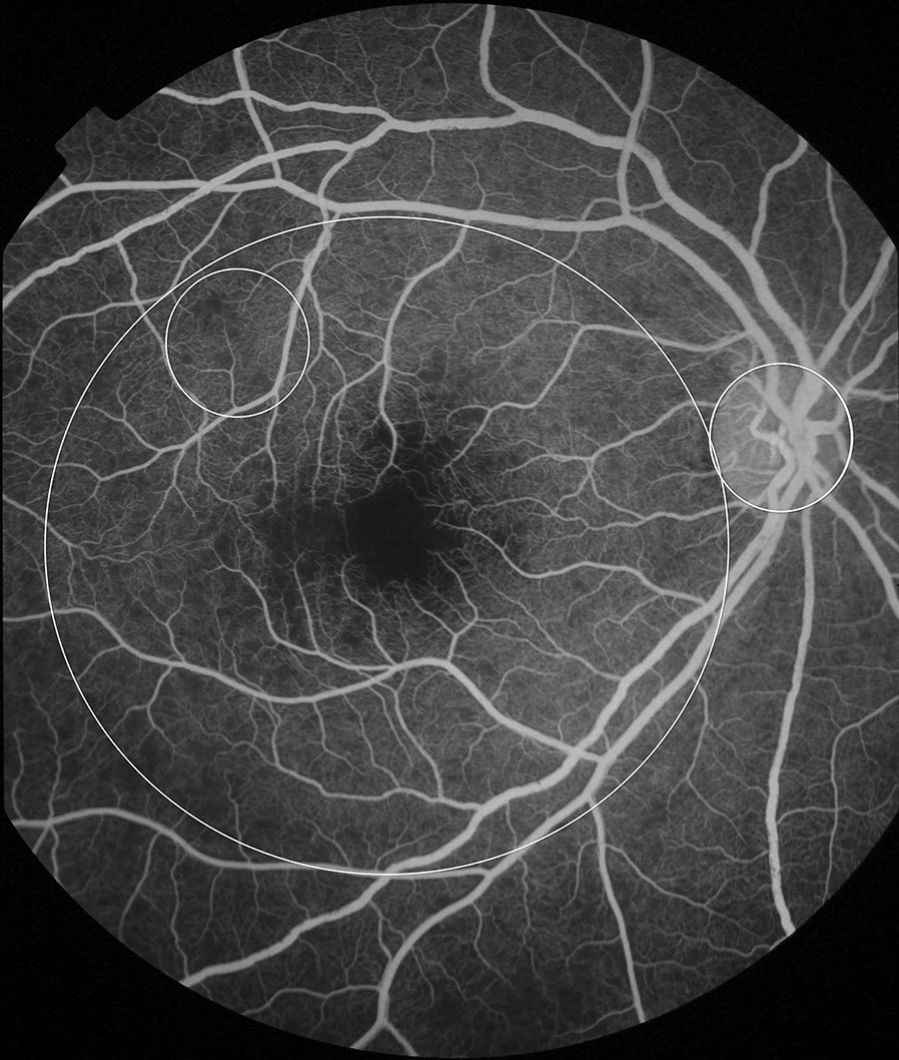
Grade 3 macular capillary non-perfusion (CNP). *Right eye* the upper limit of grade 3 macular CNP. Combined area of macular CNP is 1/3–1 disc area. Macula and one disc area are shown as *white circles*

**Fig. 11:**
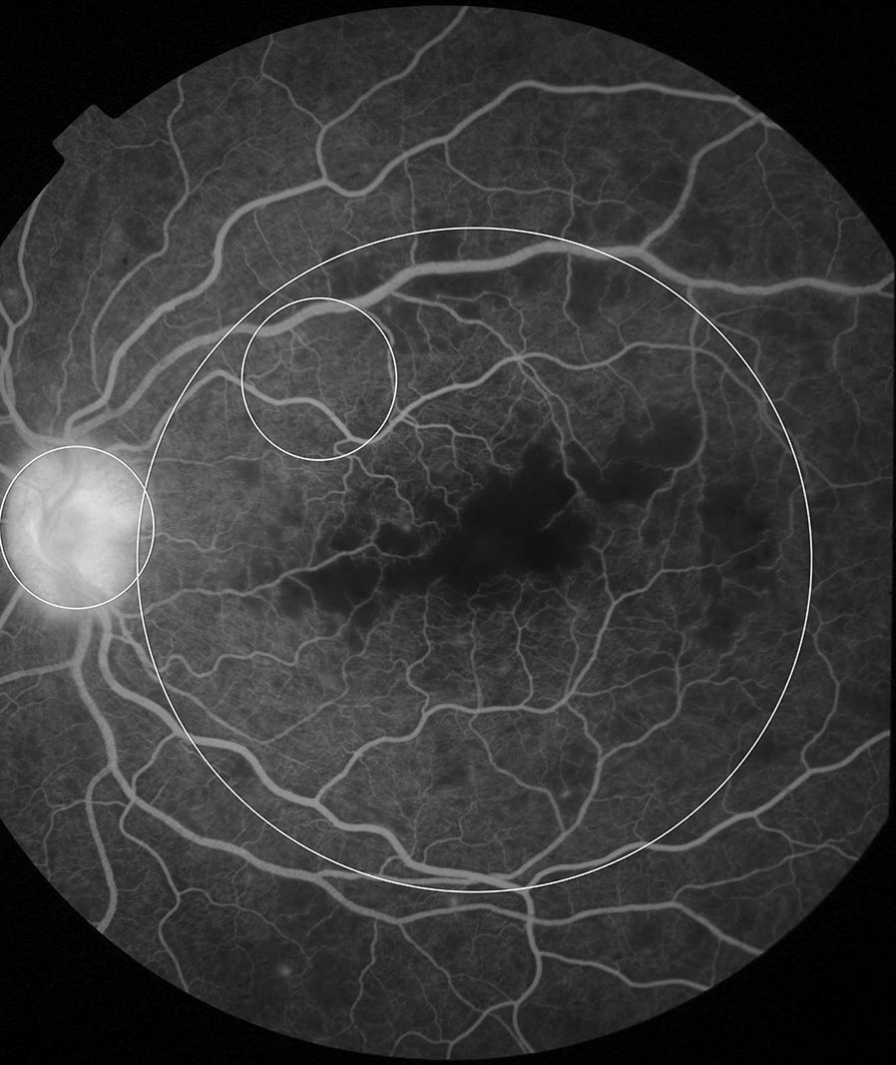
Grade 4 macular capillary non-perfusion (CNP). *Left eye* combined areas of macular CNP are >1 disc area. Macula and disc area are shown as *white circles*. Disc leak and punctate focal leak are also visible

**Fig. 12:**
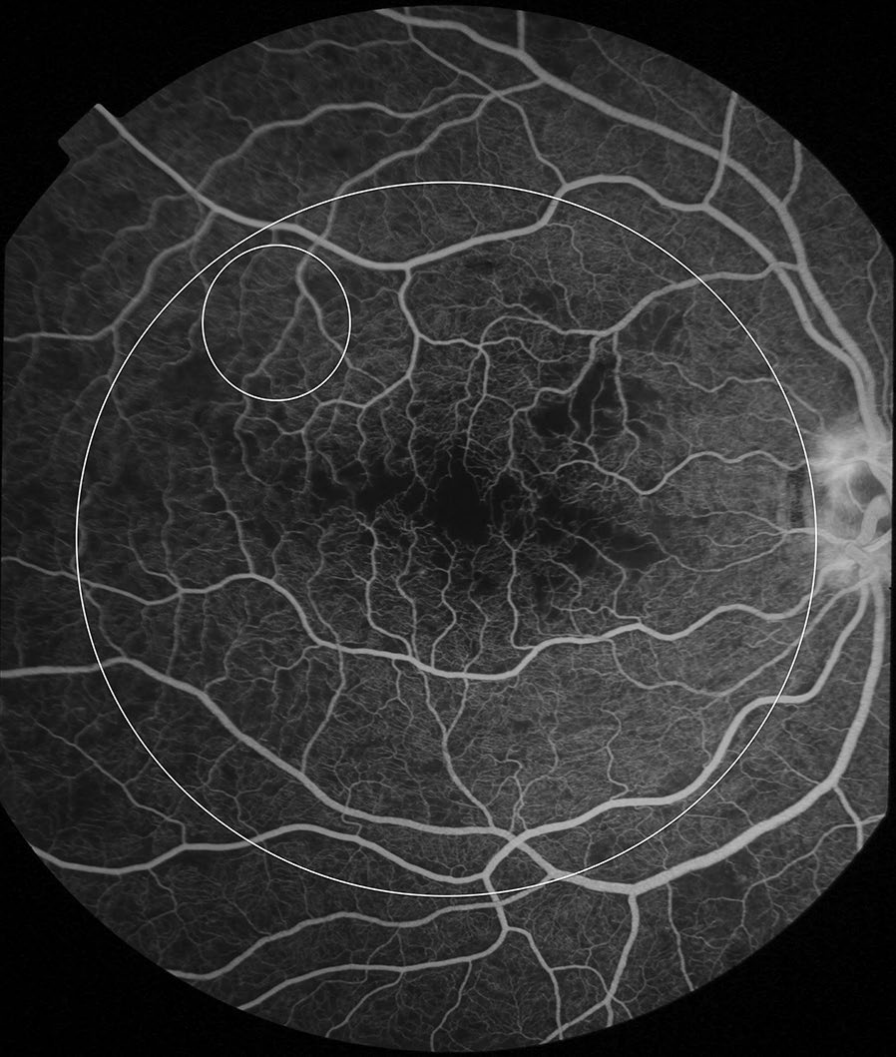
Grade 4 macular capillary non-perfusion (CNP). *Right eye* combined areas of macular CNP are >1 disc area. Macula and disc area are shown as *white circles*. Intravascular filling defects are visible in small and large venules

Macular CNP is graded on an ordinal scale (Table 4; Figs. 5, 6, 7, 8, 9, 10, 11, 12). Graders should use standard ETDRS circles to help mentally combine all CNP into the given circle [23], and should use images taken at or after the late arteriovenous phase to allow capillaries to fill completely. Grading should not include an area that notionally represents the normal foveal avascular zone (FAZ). Grade 1 macular CNP is very mild, and may capture subtle abnormalities that are not visible on indirect examination.

**Table 4:** Grades of macular capillary non-perfusion (CNP)

Lesion	Grading	Definition	Figures
Macular CNP	Cannot grade	No gradeable images of the macula exist	None
	Absent	One or more gradeable images of the macula exist, and no CNP is seen at the macula on any of these images	None
	Grade 1	A few, small areas of CNP (≥63 µm MLD) are seen, often around the foveal avascular zone	5, 6
	Grade 2	Combined area of CNP is up to ~1/3 of a disc area	7, 8
	Grade 3	Combined area of CNP is approximately 1/3–1 disc area	9, 10
	Grade 4	Combined area of CNP at the macula exceeds 1 disc area. This can be due to a few large areas or many smaller areas of CNP	11, 12

### Peripheral capillary non-perfusion (CNP)

The definition for presence of any CNP in the periphery is the same as for CNP at the macula. For peripheral CNP to be gradeable there must be at least one well-focussed image of at least one minimum area of a particular quadrant of the periphery (i.e. ≥75 % of zone 1, 2, or 3 in any quadrant). As for macular CNP, the image must be taken at or after the late arteriovenous phase. Images of the far retinal periphery may be magnified more than images of the posterior pole, owing to the optical characteristics of retinal photography done at an oblique angle through the cornea. This could potentially exaggerate the extent of CNP.

**Fig. 13:**
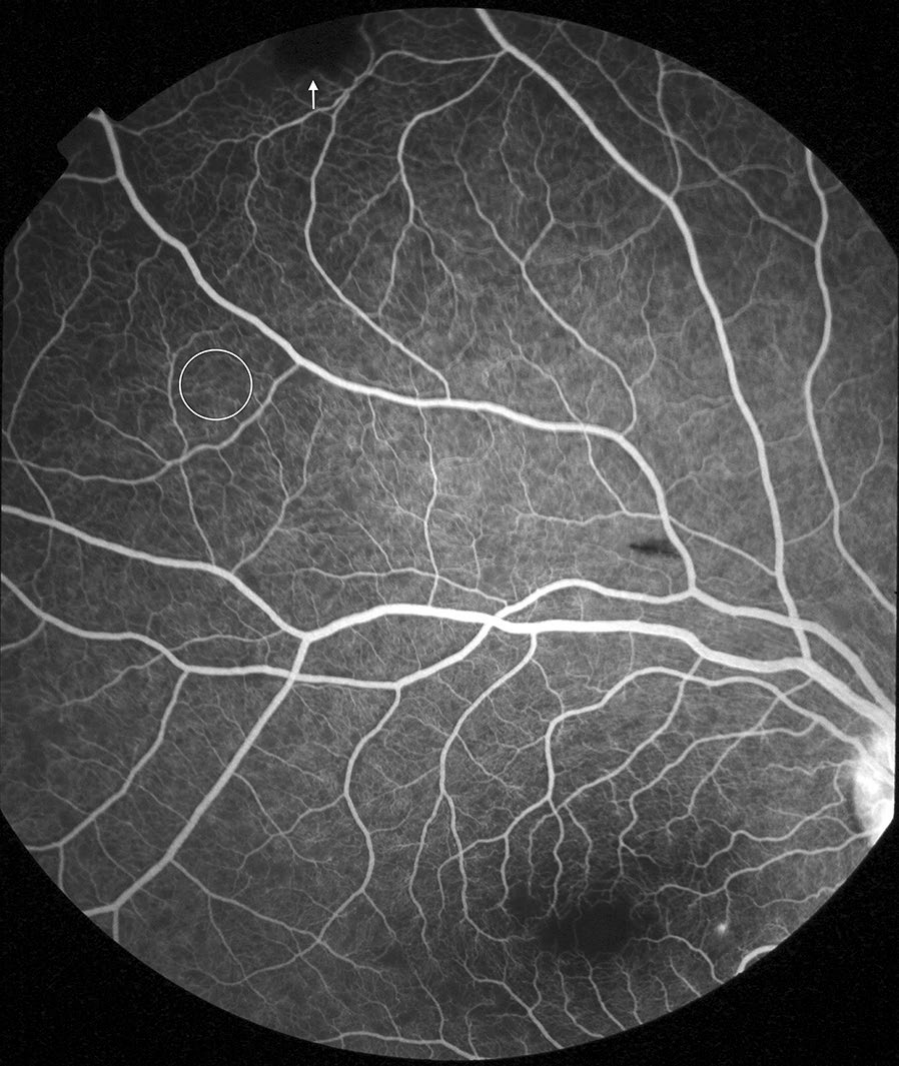
Grade 1 peripheral capillary non-perfusion (CNP). Superior retina (*right eye*). Individual areas of CNP are <1/3 disc area. 1/3 disc area is shown as a *white circle*. Note that the dark lesion (*arrow*) is a haemorrhage, and not CNP. Haemorrhage masks background fluorescence and has rounded edges, while CNP has a geographic boundary. Punctate focal leak is visible at the macula

**Fig. 14:**
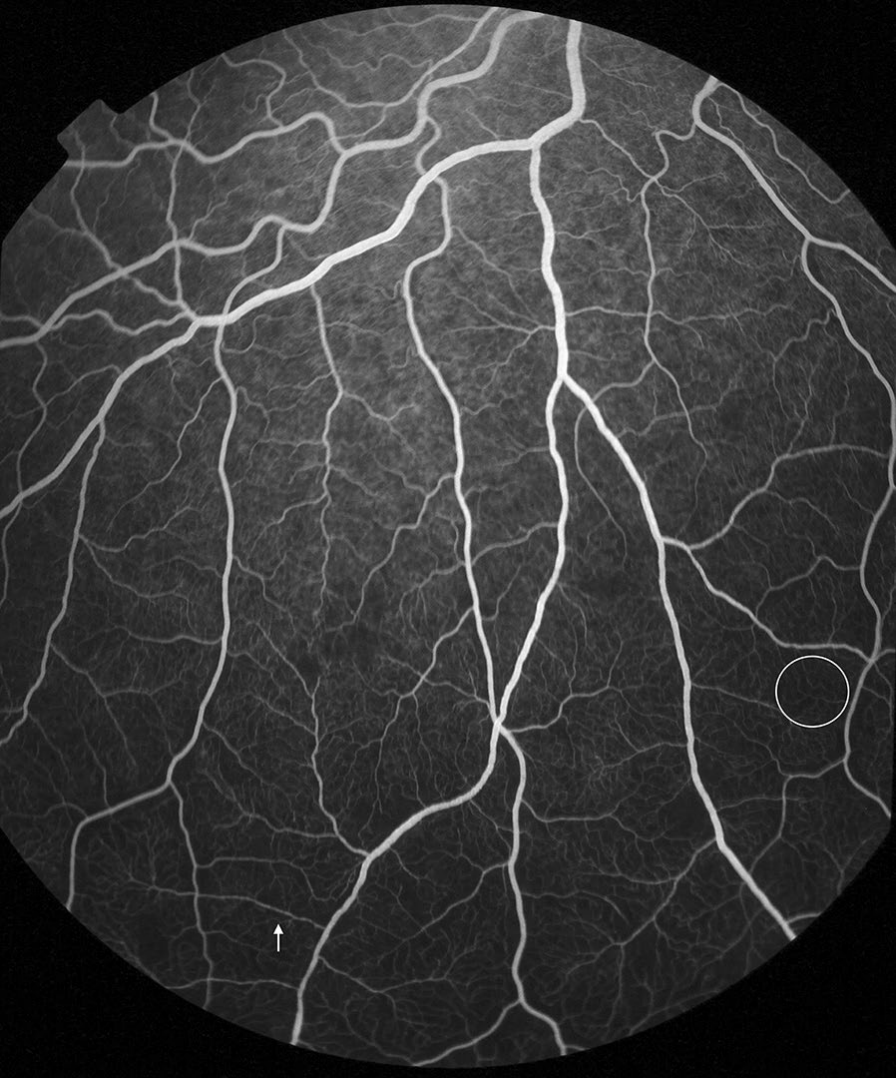
Grade 1 peripheral capillary non-perfusion (CNP). Inferior retina (*right eye*). Individual areas of CNP are <1/3 disc area, which is shown by a *white circle*. Intravascular filling defects are visible in small venules (*arrow*), and adjacent large venules

**Fig. 15:**
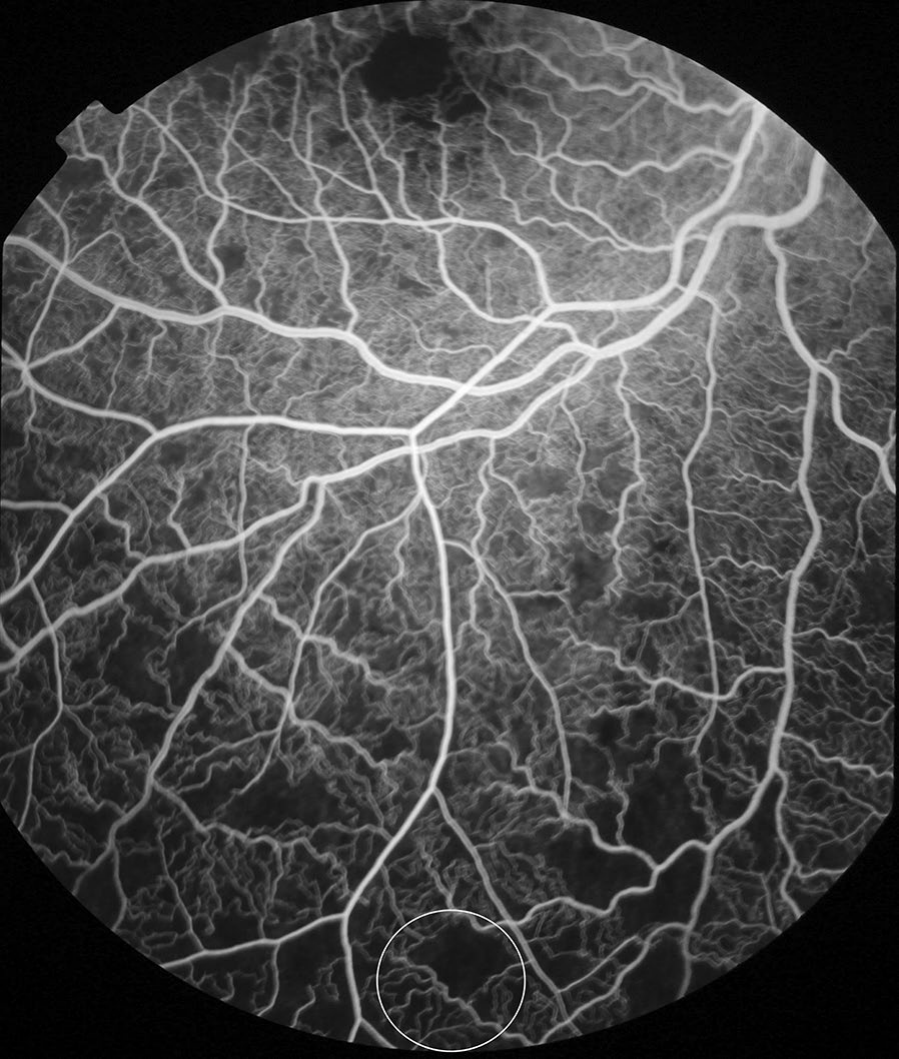
Grade 2 peripheral capillary non-perfusion (CNP). Inferior retina (*right eye*). Individual areas of CNP are between 1/3 and 1 disc area. 1 disc area is shown as a *white circle* at the *bottom of the image*. An enlarged foveal avascular zone is seen at the *top of the image*

**Fig. 16:**
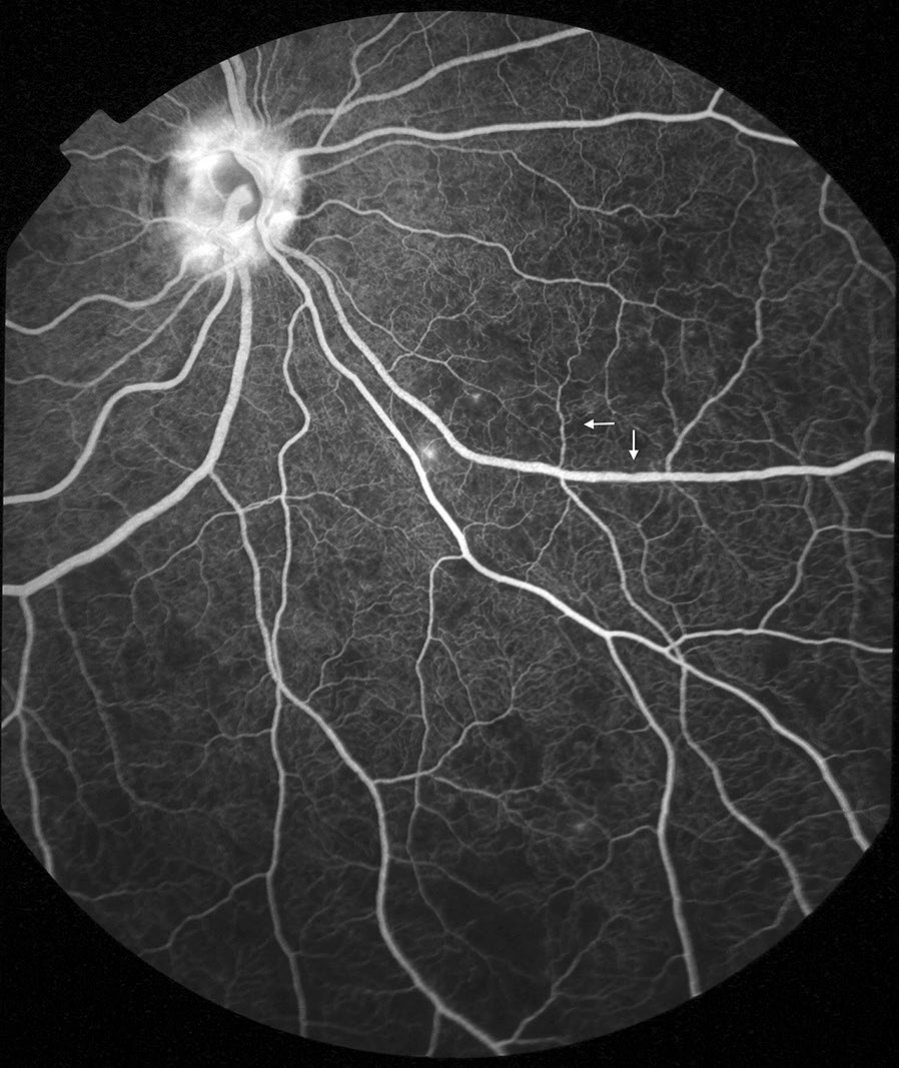
Grade 2 peripheral capillary non-perfusion (CNP). Infero-nasal retina (*right eye*). Individual areas of CNP are between 1/3 and 1 disc area. Other visible features include disc leak, punctate focal leak, and intravascular filling defects in small and large venules (*arrows*)

**Fig. 17:**
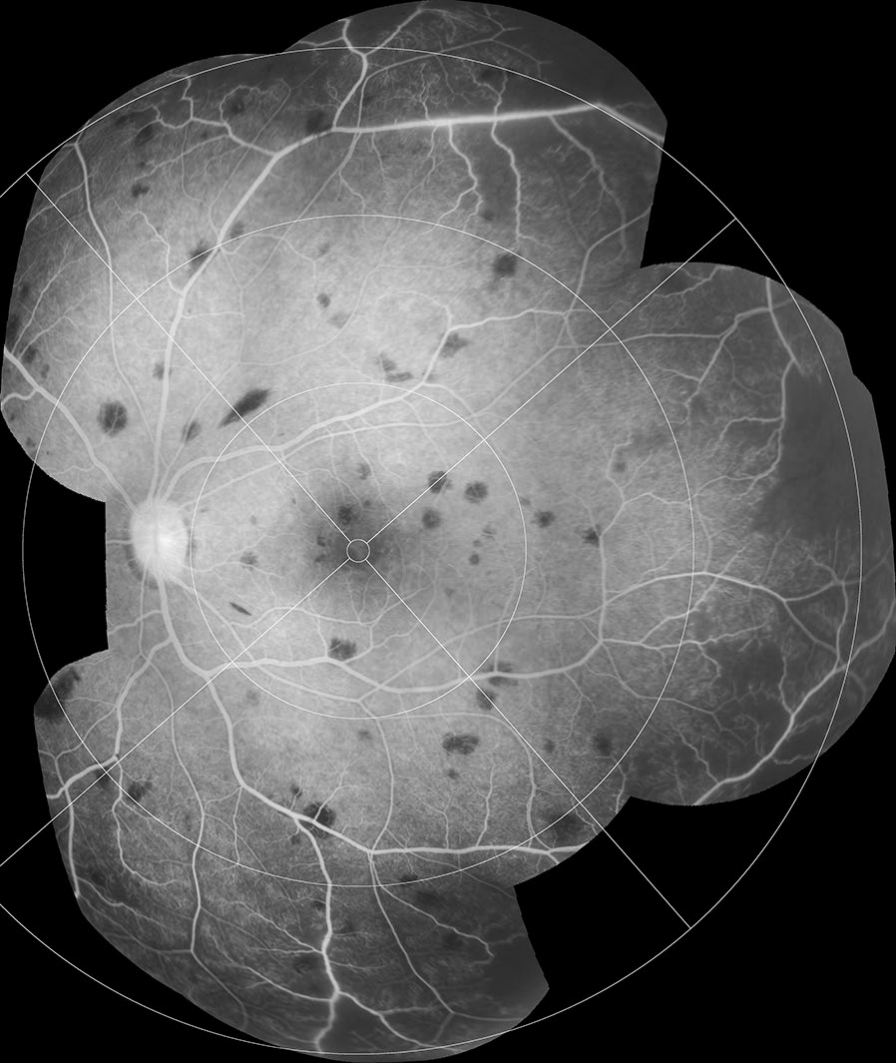
Grade 3 peripheral capillary non-perfusion (CNP). Montage of FA images (*left eye*). Individual areas of CNP are >1 disc area (superior and temporal quadrants), but do not extend into zone 2 nasally or zone 1 in other quadrants. Bays of CNP cut across large vessels and ghost vessels may be visible (e.g. temporal quadrant)

**Fig. 18:**
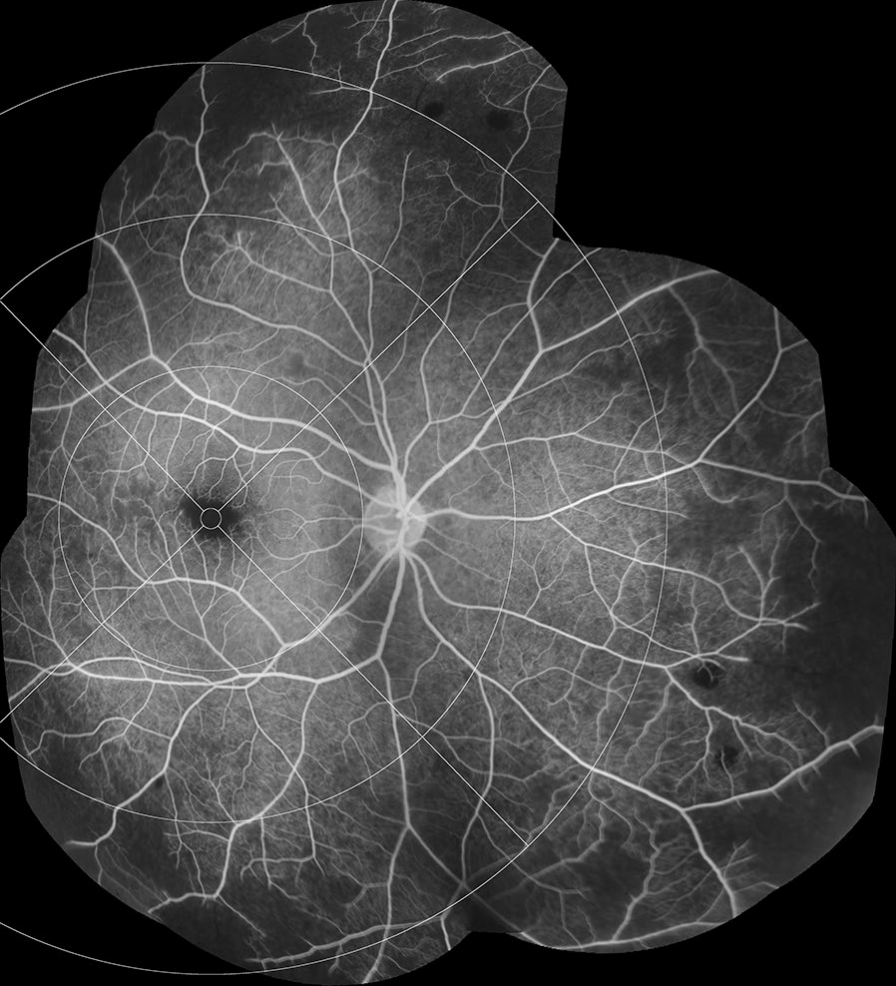
Grade 4 peripheral capillary non-perfusion (CNP). Montage of FA images (*right eye*). One or more large bays of CNP encroach on zone 1 (inferior quadrant). Bays of CNP cut across large venules and arterioles, which may appear as ghost vessels in the affected area

Peripheral CNP is graded on an ordinal scale (Table 5; Figs. 13, 14, 15, 16, 17, 18). Unlike macular CNP, peripheral CNP is graded according to the size of the largest single area of CNP, rather than the combination of all areas of CNP.

**Table 5:** Grades of peripheral capillary non-perfusion (CNP)

Lesion	Grading	Definition	Figures
Peripheral CNP	Cannot grade	No gradeable images of any retinal quadrant exist	None
	Absent	One or more gradeable images of one or more peripheral quadrants exist, and no CNP is seen on any of these images	None
	Mild grade 1	Ranges from any CNP in the periphery to multiple areas of CNP that are individually not larger than 1/3 disc area	13, 14
	Grade 2	Multiple areas of CNP that are, individually, between 1/3 and 1 disc area	15, 16
	Grade 3	Includes features of previous grades, plus one or more large bays of peripheral CNP, each >1 disc area	17
	Grade 4	One or more bays of CNP invade the superior, inferior, or temporal borders of zone 1, or zone 2 of the nasal retina	18

### Large focal leak

**Fig. 19:**
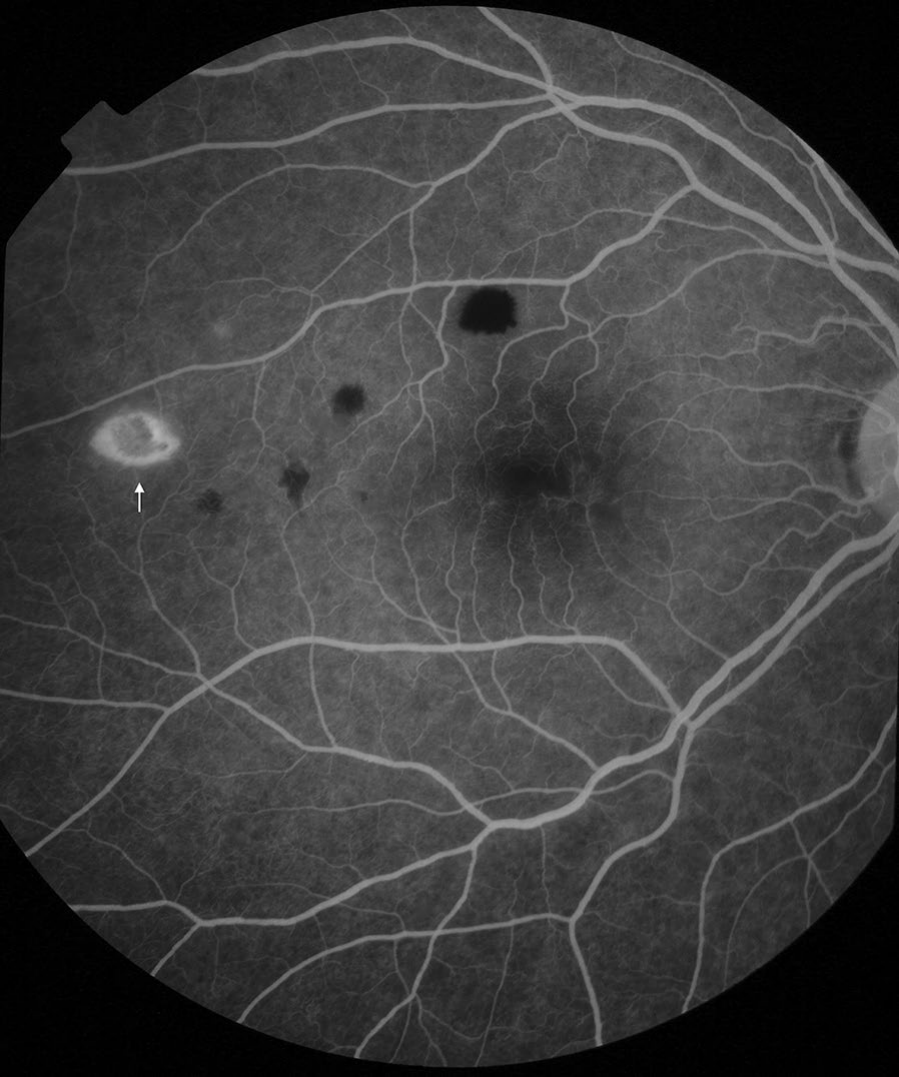
Large focal leak. *Right eye* one site of large focal leak is visible in the temporal macula (*white arrow*). Large focal leak appears to represent the evolution of retinal haemorrhage

**Fig. 20:**
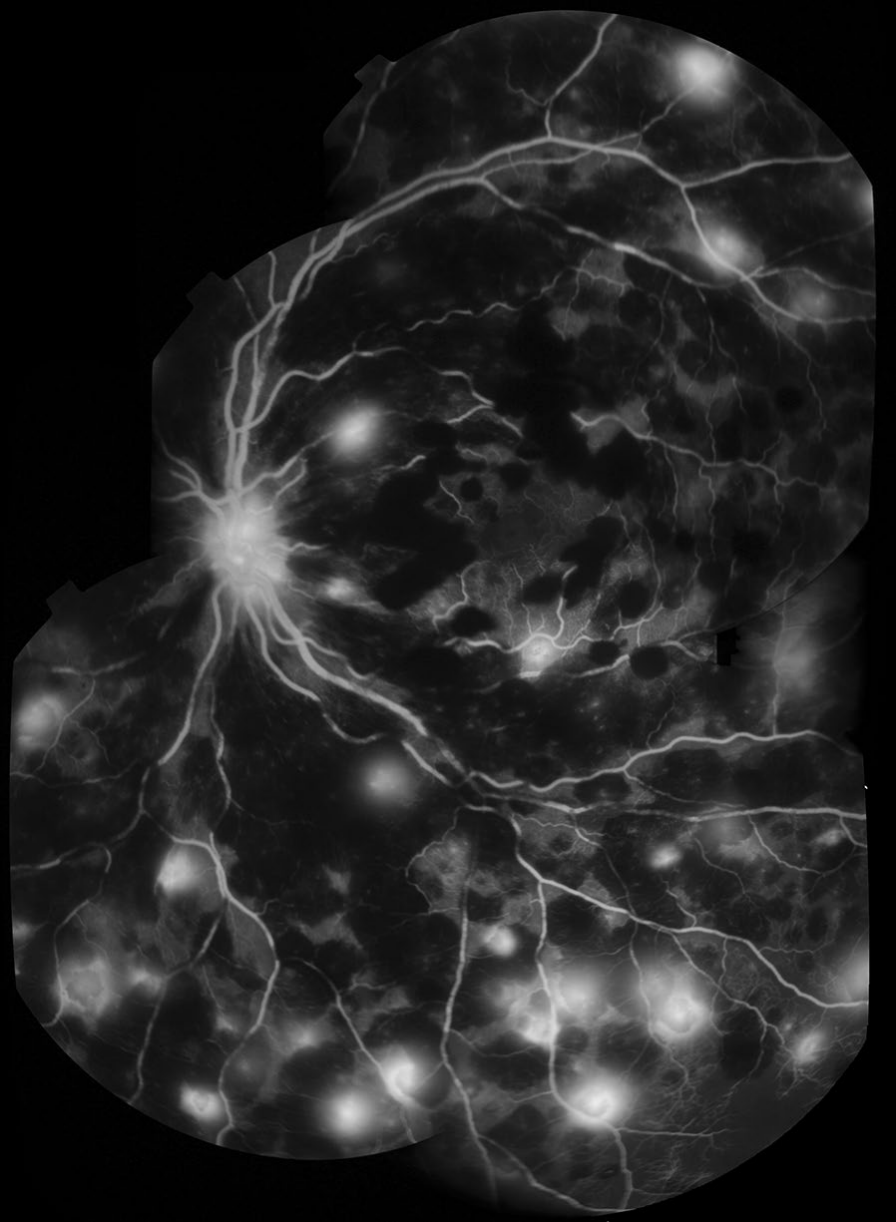
Large focal leak. Montage of FA images, *left eye*. Many sites of large focal leak are visible in the context of severe retinal haemorrhage, which causes masking of background fluorescein and the appearance of *multiple dark blots* that obscure the vasculature. Disc leak is also visible. Distinguishing sites of large focal leak can be difficult in severe cases

Large focal leak involves one or more large (>125 μm in greatest linear diameter), usually circular, areas of leak. 125 μm is approximately the width of a major venule at the optic disc. Images are rarely ungradeable for large focal leak because it is very bright. However it is probably not appropriate to grade a subject as having ‘absent’ focal leak if they do not have at least one valid image of the macula and one valid peripheral image (≥75 % of each area). Large focal leak is graded by counting each site of leak (Table 6; Figs. 19, 20).

**Table 6:** Grading large focal leak

Lesion	Grading	Definition	Figures
Large focal leak	Cannot grade	No gradeable images exist	None
	Absent	No large focal leak is seen on any gradeable image	None
	Count	A count of the number of leakage sites on a montage of fluorescein angiogram images that have been combined to illustrate the whole extent of retina captured during the fluorescein run	19, 20

### Punctate focal leak

**Fig. 21:**
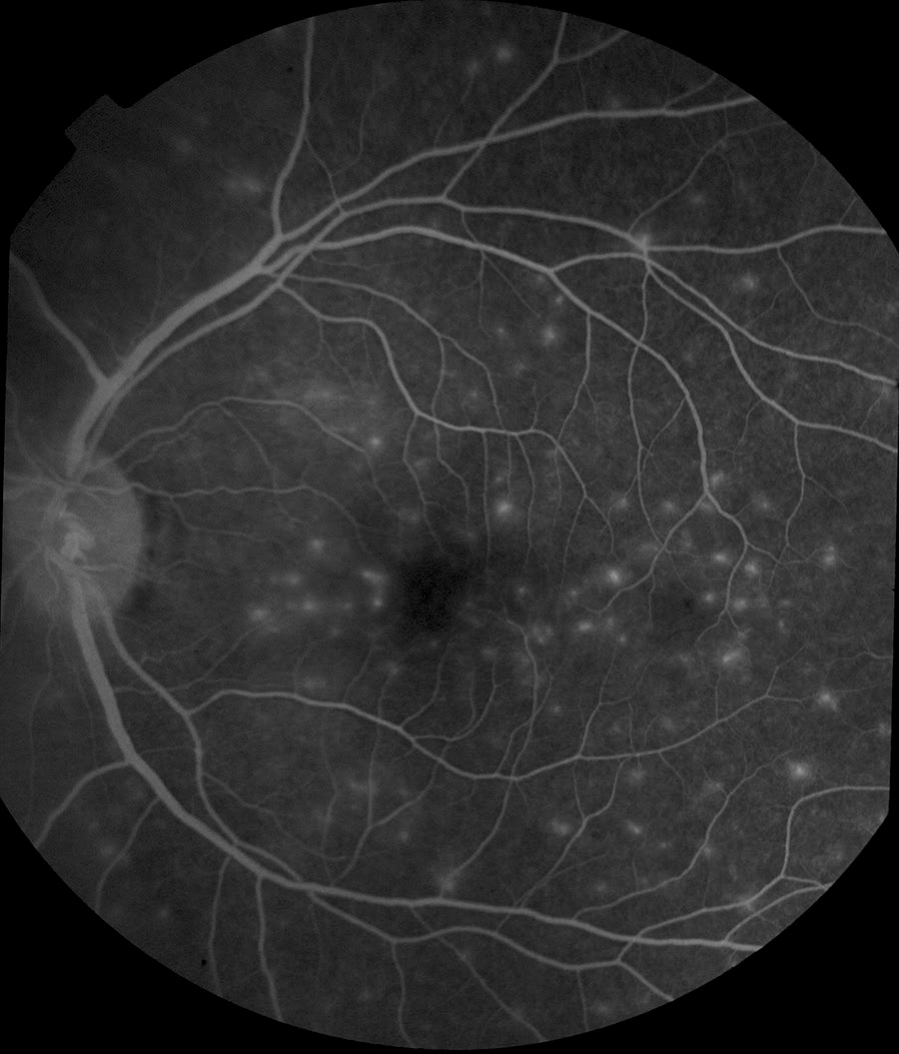
Punctate focal leak. *Left eye* multiple sites of punctate focal leak are visible

This involves small (≤125 μm) sites of leak. 125 μm is approximately the width of a major venule at the optic disc. Ideally this type of leak should be graded from a montage of images, to avoid double-counting lesions. In some cases, the very early appearance of large focal leak can look punctate, before enlarging. To avoid confusion the size of leakage foci should be checked at different times during the angiogram. Punctate focal leak is graded on an ordinal scale (Table 7; Fig. 21).

**Table 7:** Grading punctate focal leak

Lesion	Grading	Definition	Figures
Punctate focal leak	Cannot grade	No gradeable images exist	None
	Absent	No punctate focal leak is seen on any gradeable image	None
	Grade 1	1–5 sites are seen in the whole retina	7
	Grade 2	6–20 sites are seen in the whole retina	None
	Grade 3	21–50 sites are seen in the whole retina	None
	Grade 4	>50 sites are seen in the whole retina	21

### Post-capillary venule leak

**Fig. 22:**
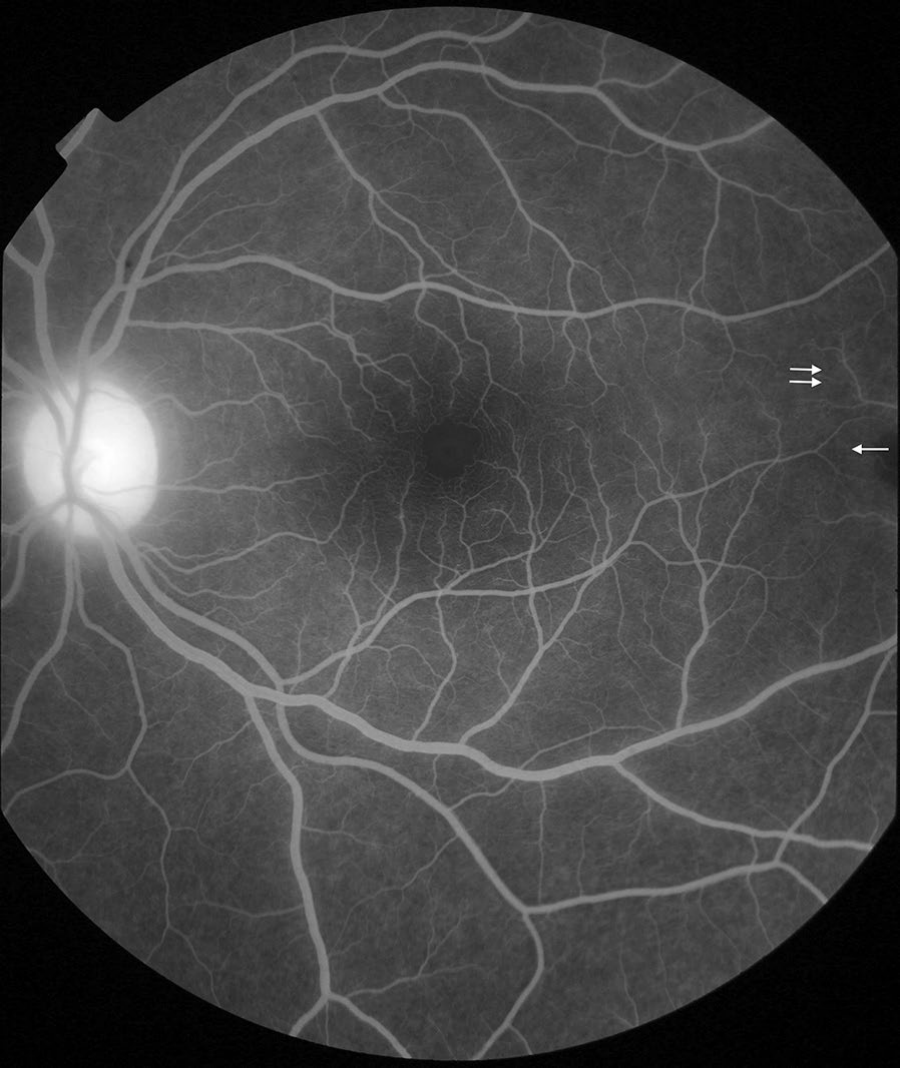
Post-capillary venule leak. *Left eye* post-capillary venule leak affecting many vessel segments. Subtle leak from venules can be detected by comparing venules (e.g. *double arrow*) with corresponding arterioles (*single arrow*). The alternating pattern of arterioles and venules makes this sign particularly clear in well focussed images of the fovea (*centre of image*). Disc leak is also visible

**Fig. 23:**
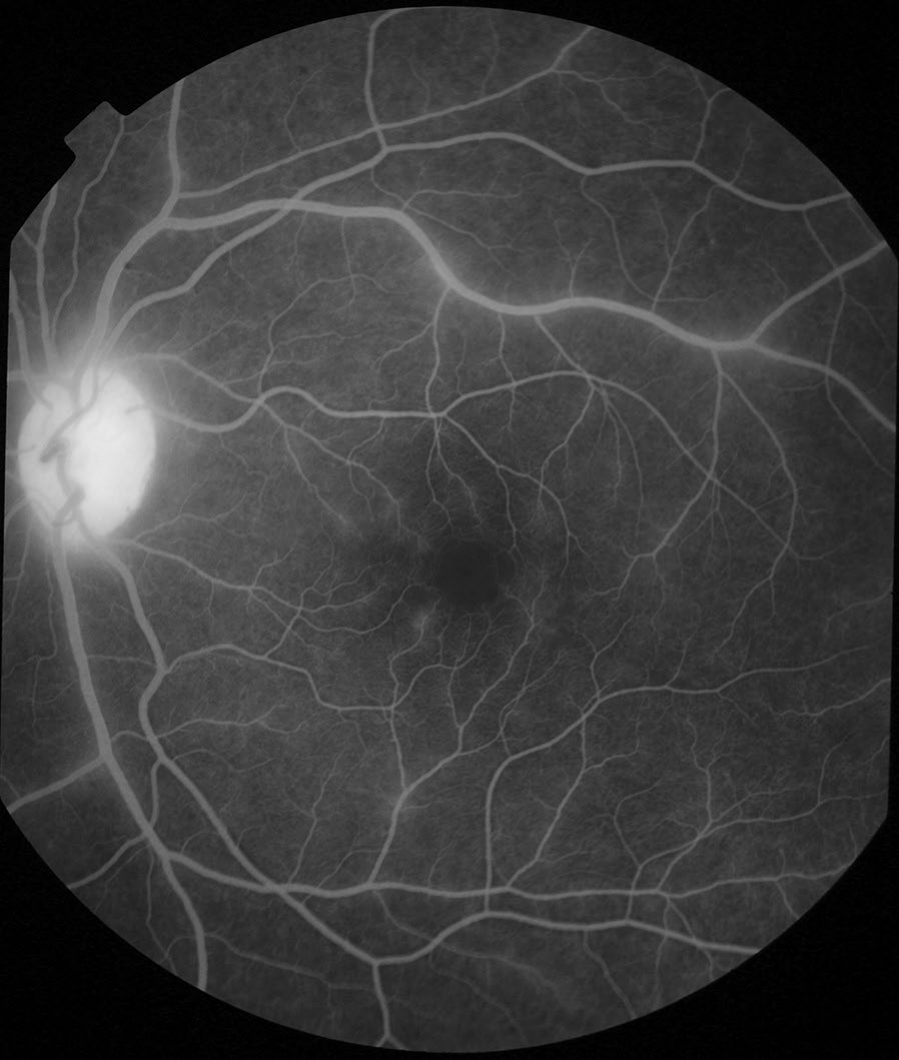
Post-capillary venule leak. *Left eye* post-capillary venule leak around the fovea. Disc leak and large/small venule leak are also visible

This describes blurring of post-capillary venules within a distance 1/3 disc diameter (500 μm) from the capillary bed (i.e. the post-capillary venule complex). When present, one or more post-capillary venules show blurring and/or increased brightness over time, compared to adjacent arterioles. Vessel segments cannot be graded for vessel leakage if adjacent vessels are not sharp enough to compare vessel margin blurring. If possible, leak should be confirmed by comparing the same vessel segments at different times during the fluorescein run. Post-capillary venule leak is graded on an ordinal scale (Table 8; Figs. 22, 23).

**Table 8:** Grading post-capillary venule leak

Lesion	Grading	Definition	Figures
Post-capillary venule leak	Cannot grade	No gradeable images exist	None
	Absent	No post-capillary venule leak is seen on any gradeable image	None
	Grade 1	1–5 post-capillary venules have leak	None
	Grade 2	6–20 post-capillary venules have leak	22, 23
	Grade 3	21–50 post-capillary venules have leak	None
	Grade 4	>50 post-capillary venules have leak	26

### Large/small venule leak

**Fig. 24:**
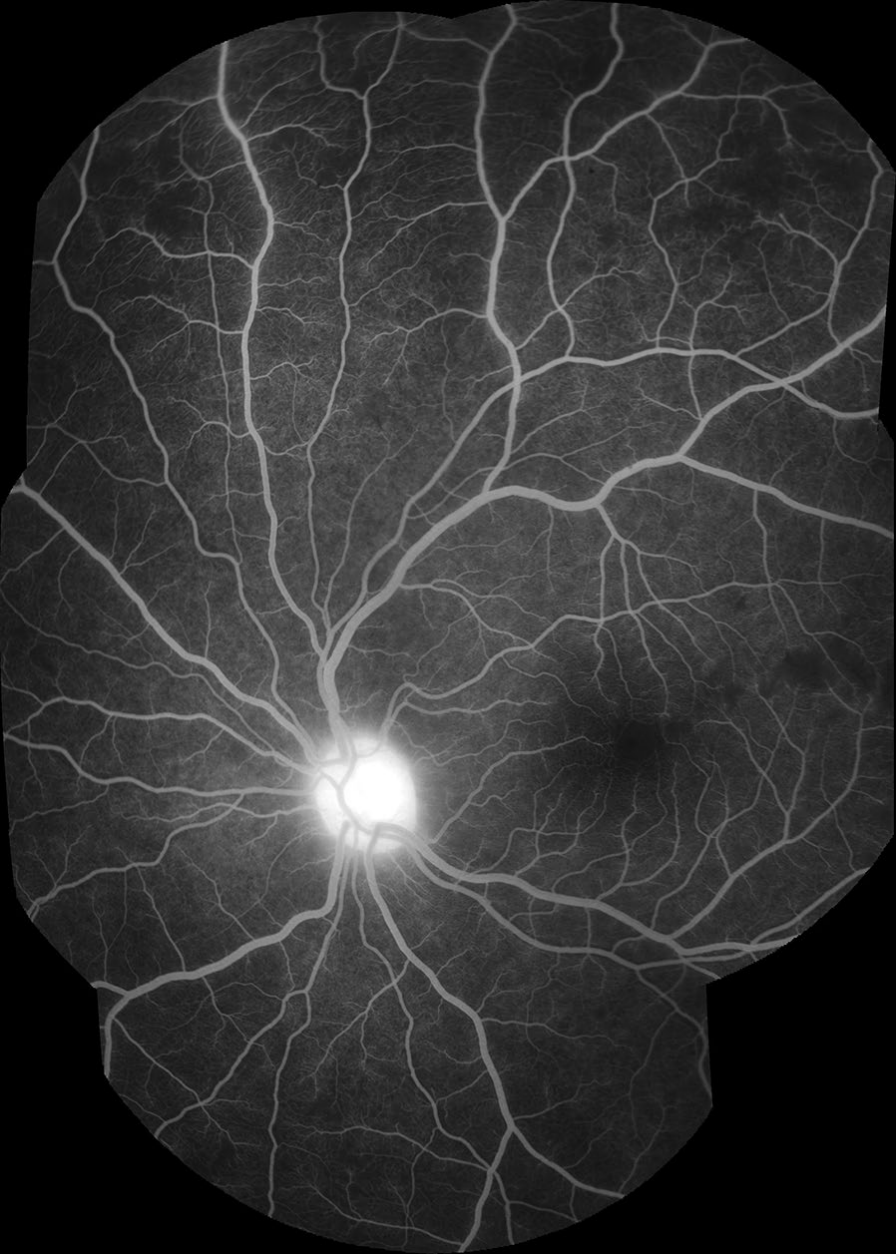
Grade 1 large/small venule leak. *Left eye* <1/3 of all large/small venule segments are leaking (superior quadrant). Disc leak is visible

**Fig. 25:**
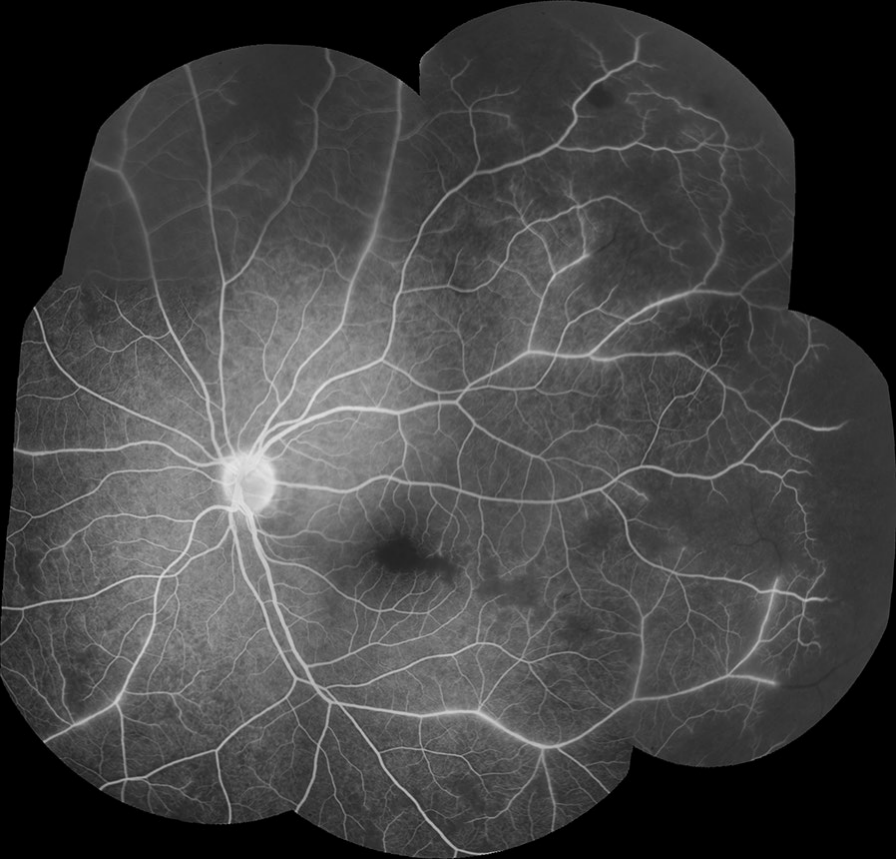
Grade 2 large/small venule leak. Montage of FA images, *left eye*. Between 1/3 and 2/3 of all large/small venule segments are leaking. Very severe macular and peripheral CNP are also visible, with ghost vessels in areas of CNP in the temporal quadrant

**Fig. 26:**
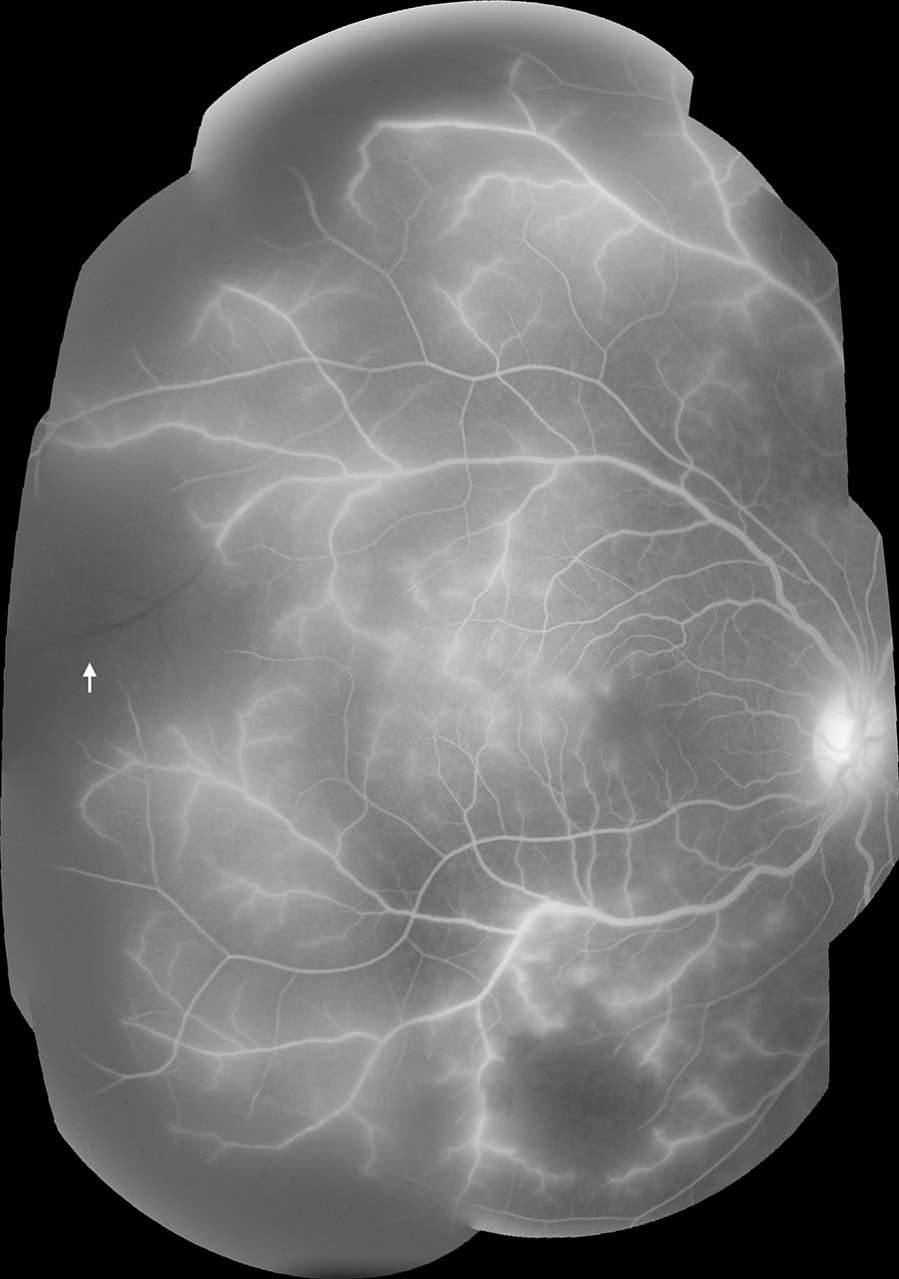
Grade 3 large/small venule leak. Montage of FA images (*right eye*). >2/3 of all large/small venule segments are leaking. Image quality is affected by extensive leakage of fluorescein. Other visible features include very severe peripheral CNP (large bays enter zone 1 in temporal and inferior quadrants. Note ghost vessel in temporal quadrant—*white arrow*), grade 4 post-capillary venule leak, and disc leak

Leakage can be inferred when the margins of venules downstream of the post-capillary venule complex are blurred, compared to adjacent arterioles. Venules may also have increased brightness over time. Ideally venule leak should be graded from a montage of images to prevent double counting of vessels. Images must be sharp enough to allow comparison of vessel margin blur between adjacent vessel segments. Large/small venule leak is graded on an ordinal scale (Table 9; Figs. 24, 25, 26).

**Table 9:** Grading large/small venule leak

Lesion	Grading	Definition	Figures
Large/small venule leak	Cannot grade	No gradeable images exist	None
	Absent	No large/small venule leak is seen on any gradeable image	None
	Grade 1	<1/3 of all small and large venule segments are blurred and/or show increased brightness over time, compared to adjacent vessel segments, for the whole retina	24
	Grade 2	1/3–2/3 of all small and large venule segments are blurred and/or show increased brightness over time, compared to adjacent vessel segments, for the whole retina	25
	Grade 3	>2/3 of all small and large venule segments are blurred and/or show increased brightness over time, compared to adjacent vessel segments, for the whole retina	26

### Disc leak

**Fig. 27:**
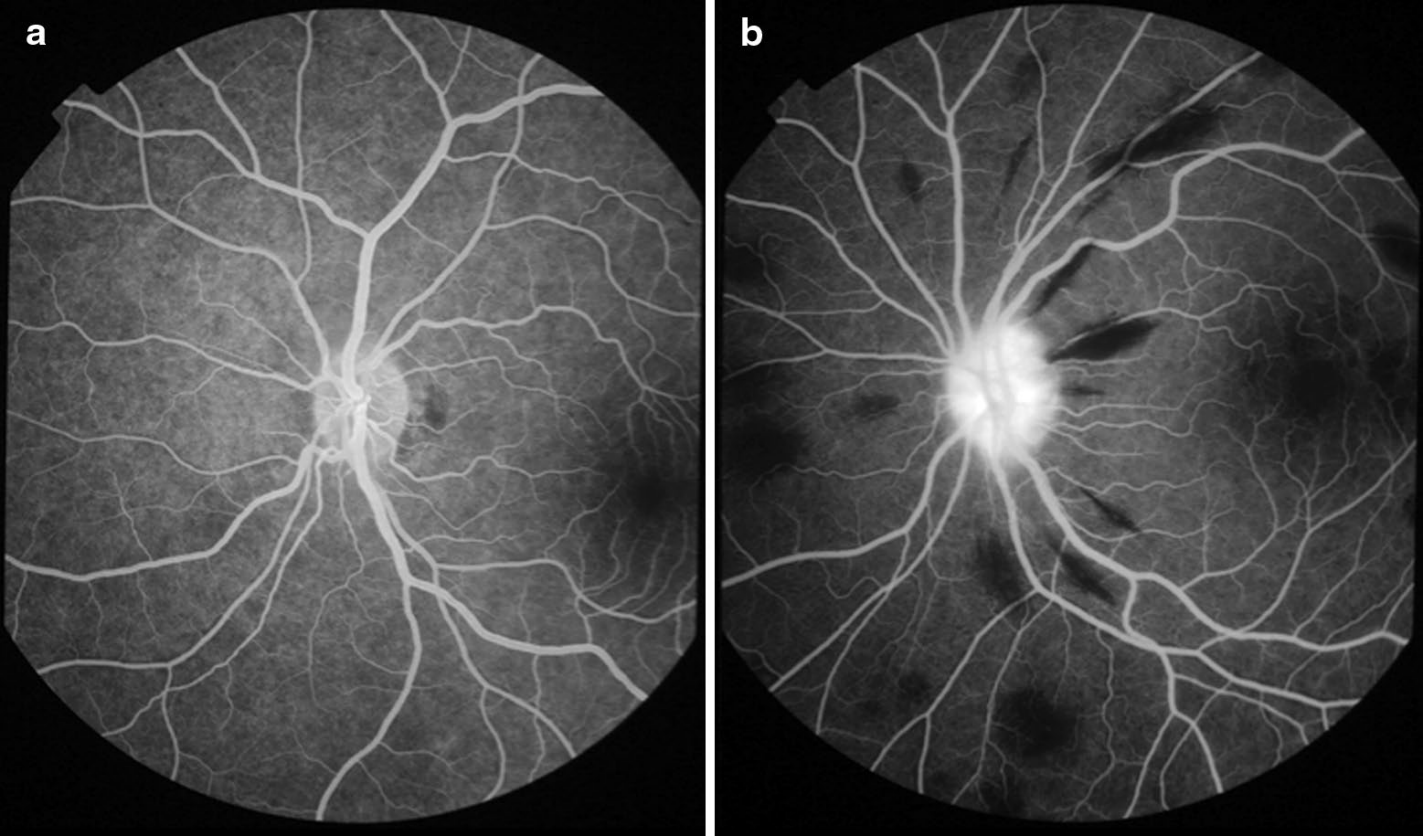
Disc leak. **a** Normal disc with no leak (7 min after injection of fluorescein). **b** Disc leak is visible as increasing brightness over time and blurring of the disc margin (2 min after injection). Peripapillary haemorrhages are seen as *black flame-shaped* lesions, due to masking of background fluorescence

Disc leak is defined as an increase in brightness with blurring of the disc margin, over time during the fluorescein run. It can only be graded ‘absent’ by examination of images taken during mid to late phases of the fluorescein run. If no mid or late images exist, or if the disc is obscured, it should be graded ‘CG’ (Table 10; Fig. 27).

**Table 10:** Grading disc leak

Lesion	Grading	Definition	Figures
Disc leak	Cannot grade	No gradeable images exist	None
	Absent	No disc leak is seen on any gradeable image	27
	Present	Disc leak is present on one or more gradeable images	27

### Intravascular filling defects (IVFD)

**Fig. 28:**
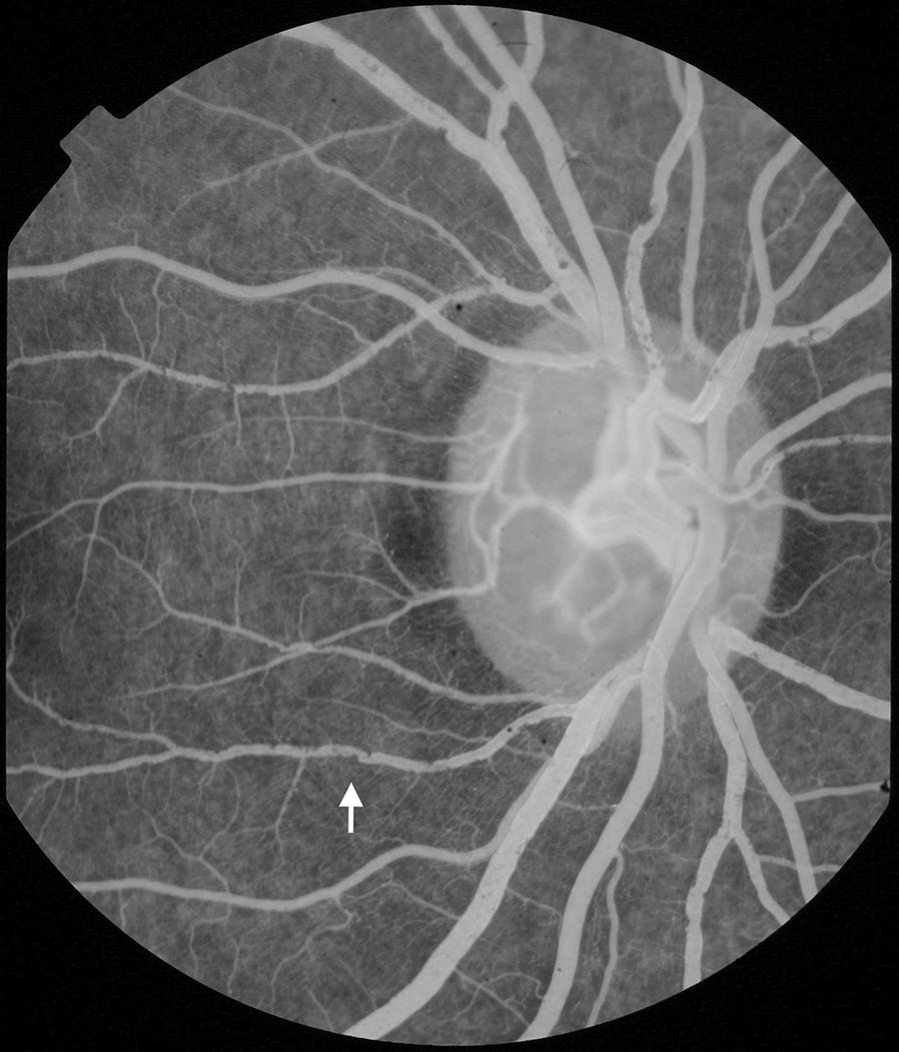
Intravascular filling defects (IVFD). 20° image (*right eye*). In this image IVFD are prominent in vessels at the disc (*white arrow*). The venules appear to be affected much more severely than corresponding arterioles

**Fig. 29:**
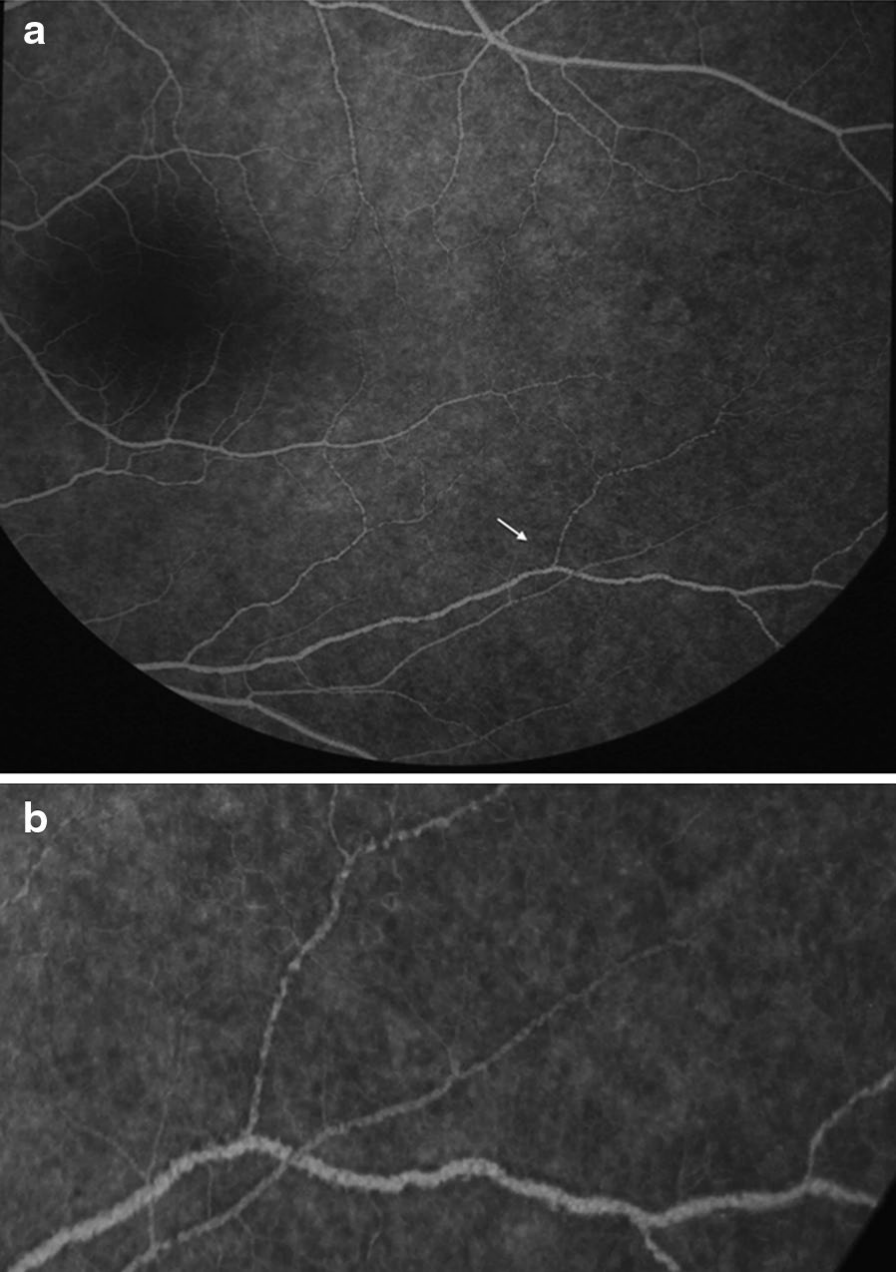
Intravascular filling defects (IVFD)**. a** *Left eye* 50º image of the macula and temporal periphery. IVFD are prominent in venules and arterioles. **b** Full size image of the vessel junction marked in **a** (*white arrow*), illustrates mottling of the blood column

Intravascular filling defects are abnormalities of the blood column. They can occur in large and small retinal vessels of all types but appear to be most frequent and dense in post-capillary venules [17]. In larger vessels the appearance can range from slight irregularity to the impression that small ‘bites’ have been taken from the vessel. Small vessels can appear mottled. The observation of IVFD depends on being able to discern abnormal texture in vessels. This is not possible in images that are blurred, or where vessels are obscured by haemorrhage or leak. It may be possible to see severe IVFD in slightly blurred images, but confidently ruling out the presence of any defects requires images sharp enough to identify individual capillaries next to the vessels being graded. If image quality is poorer than this, filling defects cannot be ruled out, and the images should be graded ‘CG’ (Table 11; Figs. 28, 29).

**Table 11:** Grading intravascular filling defects (IVFD)

Lesion	Grading	Definition		Figures
Intravascular filling defects (IVFD)	Cannot grade	No gradeable images exist (adjacent capillaries are not clear). CG can be assigned to one or more vessel types		None
	Capillaries	Absent	IVFD are not seen in any capillaries	None
		Present	IVFD are seen in capillaries	None
	Post-capillary venule complex	Absent	IVFD are not seen in any post-capillary venules	None
		Present	IVFD are seen in post-capillary venules	None
	Pre-capillary arteriole complex	Absent	IVFD are not seen in any pre-capillary arterioles	None
		Present	IVFD are seen in pre-capillary arterioles	None
	Small venules	Absent	IVFD are not seen in any small venules	None
		Present	IVFD are seen in small venules	28, 29
	Small arterioles	Absent	IVFD are not seen in any small arterioles	None
		Present	IVFD are seen in small arterioles	28, 29
	Large venules	Absent	IVFD are not seen in any large venules	None
		Present	IVFD are seen in large venules	28, 29
	Large arterioles	Absent	IVFD are not seen in any large arterioles	None
		Present	IVFD are seen in large arterioles	28, 29

### Other features

Other features besides those described above may be seen on FA in severe malaria. These include cystoid macular oedema and leakage from arterioles. If signs that are not part of the standardized grading scheme are observed this should be indicated on the grading form, with a description of the lesion.

## Performance of the grading scheme

Between 2006 and 2014, 285 children had FA imaging of the left eye on admission to the Research Ward. The clinical characteristics of this group are listed in Table 12.

**Table 12:** Clinical characteristics of 285 subjects with admission fluorescein angiogram

	Median	25th quartile	75th quartile	Number	Percent
Total	n/a	n/a	n/a	285	100.00
History					
Age	39.00	28.00	58.50	285	100.00
Gender					
Male				137	48.07
Female				148	51.93
Diagnosis					
CM				124	43.51
SMA				1	0.35
CM + SMA				152	53.33
Other malarial diagnosis				4	1.40
Non-malarial illness				4	1.40
Examination					
Weight (kg)	12.00	10.00	15.00	285	100.00
Height (cm)	93.00	84.00	104.00	281	98.60
Mid-upper arm circumference (cm)	15.00	14.50	16.00	282	98.95
Temperature (rectal, °C)	38.80	38.10	39.50	285	100.00
Heart rate (beat/s)	150.00	133.50	169.00	285	100.00
Systolic blood pressure (mmHg)	97.00	90.00	106.00	268	94.04
Respiratory rate (breaths/s)	44.00	36.00	52.00	285	100.00
Respiratory distress					
Absent				195	68.42
Present				90	31.58
Blantyre Coma Score					
0				20	7.02
1				130	45.61
2				135	47.37
Retinopathy^a^ (either eye)					
Absent				21	7.37
Present				264	92.63
Investigations					
Peripheral parasitaemia (parasites/μl)	45,000.00	2535.50	184,000.00	277	97.19
White cell count (cells)	10,050.00	7200.00	14,500.00	270	94.74
Platelets (platelets)	62,500.00	33,000.00	105,000.00	268	94.04
Haematocrit (%)	20.00	16.00	24.85	282	98.95
Venous lactate (mmol/L)	4.80	2.90	9.00	281	98.60
Plasma HRP2 (ng/ml)	7405.00	3117.00	10,452.00	263	92.28
HIV status					
Negative				224	78.60
Positive				39	13.68
Outcome					
Full recovery				213	74.74
Sequelae				36	12.63
Death				36	12.63

^a^Retinopathy assessed by dilated bilateral indirect ophthalmoscopy

Dual grading with adjudication was performed on 285 left eye image sets from these subjects. The great majority of FA images in this sample were from children with CM with or without severe malarial anaemia (276/285, 96.84 %). Similarly, the majority had malarial retinopathy visible on ophthalmoscopic exam (264/285, 92.63 %). A minority (5/285) had other severe malarial syndromes, or non-malarial illness (4/285). The distribution of FA features for these groups is shown in Additional file 2. The data in Additional file 2 are taken from the adjudicated grading for the left eye.

The most common FA feature in retinopathy-positive CM was CNP. Some level of macular CNP was seen in all cases, and peripheral CNP was seen in ~95 %. Grade 1 macular CNP is extremely mild, and may well be present even when macular whitening is not visible with indirect ophthalmoscopy. However ~80 % of retinopathy-positive CM cases had grade 2 CNP or above. Of the leakage types, disc leak was seen in ~80 % of retinopathy-positive CM cases, post-capillary venule leak in ~50 %, Large venule leak in ~60 %, punctate focal leak in ~30 %, and large focal leak in ~12 %. Venular IVFD were seen in 60–90 % of retinopathy-positive CM cases, and arteriolar IVFD in 8–40 % (Additional file 2).

Grading scores were compared between grader 1 and grader 2 for all left eye images. Agreement was >80 % for six features, 70–80 % for three features, 60–69 % for two features, and 50–59 % for three features. The lowest levels of agreement were found for IVFD in capillaries, small arterioles, and large arterioles (Table 13).

**Table 13:** Inter-grader agreement (left eye)

Variable	Number with data for comparison	Frequency of feature (grader 1)	Observed agreement between graders (%)
Macular CNP	248	266/268	74.9
Peripheral CNP	264	263/278	80.40
Punctate focal leak	273	77/281	89.10
Disc leak	278	237/283	82.73
Post capillary venule leak	273	115/275	84.25
Large venule leak	279	88/280	71.92
Large focal leak^a^	285	34/285	95.95
Capillary IVFD	212	62/220	59.91
Post-capillary venule unit IVFD	231	212/237	75.32
Pre-capillary arteriole unit IVFD	235	52/243	67.23
Small venule IVFD	265	240/267	84.91
Small arteriole IVFD	263	55/267	57.79
Large venule IVFD	265	167/268	69.43
Large arteriole IVFD	264	32/268	59.47

*CNP* capillary non-perfusion, *IVFD* intravascular filling defects

^a^Large focal leak is a count variable with skewed distribution. Agreement estimated for categories “absent”, “1 site of leak”, “>1 site of leak”

## Discussion

A scheme for grading FA images of malarial retinopathy was developed and tested, along with example images to aid future interpretation of FA in this disease. This scheme can reliably classify dysfunction in the retinal vasculature seen in paediatric CM, in spite of challenges around image acquisition and quality that do not apply to most other retinal conditions. Unlike patients with diabetic retinopathy or age-related macular degeneration, patients with severe malaria are acutely unwell and capturing high quality images is difficult. Although this scheme was tested on images from children with CM, this will be applicable to future studies of severe malaria in general since malarial retinopathy is seen in some severe malarial syndromes other than CM, and in adults as well as children.

### Angiographic features in paediatric severe malaria

FA features were commonly seen in children with retinopathy-positive CM, and could range from mild to severe. The same features were sometimes observed in children with other diagnoses (Additional file 2). This is consistent with previous observations of malarial retinopathy in severe non-cerebral, moderate, and uncomplicated paediatric malaria [7]. Cases without obvious retinopathy on ophthalmoscopic exam, or with diagnoses other than malaria, were included for completeness and to ensure the maximum number of cases for assessment of the grading scheme—which was the primary reason for this analysis. The number of subjects with non-CM diagnoses is not large enough to draw conclusions about the frequency of FA signs in these groups in the population in general, but the results indicate that retinal abnormalities do exist in patients without other signs of malarial retinopathy, and also in patients with diagnoses other than malaria. Increasingly sensitive retinal imaging modalities may potentially reveal previously unnoticed retinal dysfunction, both in CM and other neurological infections.

### Inter-grader agreement

Good levels of agreement were found between graders for the majority of features (Table 13), including CNP (75–80 %) and four of the five types of leakage (>83 %). Grading of large venule leak had an agreement of 72 %, and may have been affected by variable focus, brightness, and clarity during the FA run. Graders should review the full run of images to aid in this interpretation. Grading IVFD was more problematic, especially for the capillary network. Although they can be seen clearly on some exceptionally sharp images, experience suggests that capillary IVFD are probably beyond the resolution of standard FA, and that this feature should be treated with caution. Other existing or emerging imaging modalities may be more suitable for studying retinal capillaries in CM. Agreement for IVFD was consistently lower on the arteriolar compared to the venular side (57–67 vs 69–85 % respectively). This may be a result of bleaching secondary to a greater concentration of fluorescein in arterioles compared to venules. Nevertheless, expert adjudication of inter-grader discrepancies for arteriolar IVFD was much more feasible than for capillary IVFD and should provide reasonable quality data.

Disagreements between graders are inevitable and occur in most, if not all, medical imaging quantified by human observers. Adjudication of dual grading is an accepted approach to improving validity of grading systems and is widely used in research settings. Careful attention to training and quality control, and inclusion of adjudication gives us good confidence that this scheme can reliably be used to assess the FA features of malarial retinopathy. Automated grading techniques based on computerized image analysis may provide more reliable quantification than manual grading in the future [25].

### Using this grading scheme in future studies of malarial retinopathy

In order to maximize the quality of grading data, studies of malarial retinopathy should ensure that images are taken by experienced retinal photographers and graded by observers who are familiar with grading retinal images. Ideally multiple graders should develop a mutual consensus on feature recognition by training on a set of malarial retinopathy test images before starting formal grading. The authors recommend that future studies of retinal imaging in malaria also perform dual grading with independent adjudication. Studies on malarial retinopathy should describe photographer and grader experience. Data on the overall quality of images and amount of retinal periphery captured give useful indications about the robustness of grading data in a study, and it is suggested that these should be reported along with the frequency of specific FA features. These recommendations are not specific to FA images, but are applicable to collection and grading colour images and other retinal imaging modalities that may yet be applied to severe malaria.

## Conclusions

This FA grading scheme offers a practical and consistent method to investigate malarial retinopathy in different populations and allow comparison between studies. This formal grading scheme will enhance understanding of severe malaria by allowing direct comparison of angiographic findings in malarial retinopathy between children and adults, and between patients in different geographical areas. The reliability of grading is feature specific, and is also likely to depend on overall image quality and grader experience. Good levels of agreement can be achieved with experienced graders. Independent adjudication should be used to maximize grading accuracy.

## Additional files

**Additional file 1.** Fluorescein angiography in severe malaria: grading form.
**Additional file 2.** Fluorescein angiogram features in 285 subjects with admission angiogram of the left eye, reported by clinical diagnosis. Table of angiographic features in subjects with different diagnoses, with and without observable malarial retinopathy on dilated indirect ophthalmoscopy pre-angiogram.

## Abbreviations

CG: cannot grade
CM: cerebral malaria
CNP: capillary non-perfusion
DA: disc area
DD: disc diameter
ETDRS: Early Treatment Diabetic Retinopathy Study research group (1985)
FA: fluorescein angiogram or fluorescein angiography
FAZ: foveal avascular zone
IVFD: intravascular filling defects
