# Supplementary material for: Grading fluorescein angiograms in malarial retinopathy
Source: Malar J. 2015 Sep 24;14:367. doi: 10.1186/s12936-015-0897-7 (PMC4583163; doi:10.1186/s12936-015-0897-7)
Supplement: Supplementary file 2 — Additional file 2. Fluorescein angiogram features in 285 subjects with admission angiogram of the left eye, reported by clinical diagnosis. Table of angiographic features in subjects with different diagnoses, with and without observable malarial retinopathy on dilated indirect ophthalmoscopy pre-angiogram. [file 12936_2015_897_MOESM2_ESM.docx]

|  | Retinopathy negative | | | | Retinopathy positive | | | | |
| --- | --- | --- | --- | --- | --- | --- | --- | --- | --- |
| Diagnosis | Cerebral malaria | Cerebral malaria and severe malarial anaemia | Other malaria diagnosis | Non-malarial diagnosis | Cerebral malaria | Severe malarial anaemia | Cerebral malaria and severe malarial anaemia | Other malaria diagnosis | Non-malarial diagnosis |
| Number with FA images of the left eye | 15 | 2 | 1 | 3 | 109 | 1 | 150 | 3 | 1 |
|  | Number (%) of subjects with each grade of feature | | | | | | | | |
| Macular CNP | | | | | | | | | |
| Absent | 0 (0) | 1 (50.00) | 0 (0) | 0 (0) | 0 (0) | 0 (0) | 0 (0) | 0 (0) | 0 (0) |
| Grade 1 | 7 (46.67) | 0 (0) | 0 (0) | 2 (66.67) | 15 (13.76) | 0 (0) | 25 (16.67) | 0 (0) | 0 (0) |
| Grade 2 | 7 (46.67) | 1 (50.00) | 1 (100) | 1 (33.33) | 57 (52.29) | 0 (0) | 67 (44.67) | 2 (66.67) | 1 (100) |
| Grade 3 | 0 (0) | 0 (0) | 0 (0) | 0 (0) | 21 (19.27) | 0 (0) | 34 (22.67) | 0 (0) | 0 (0) |
| Grade 4 | 0 (0) | 0 (0) | 0 (0) | 0 (0) | 9 (8.26) | 0 (0) | 17 (11.33) | 0 (0) | 0 (0) |
| Cannot grade | 1 (6.67) | 0 (0) | 0 (0) | 0 (0) | 7 (6.42) | 1 (100) | 7 (4.67) | 1 (33.33) | 0 (0) |
| Peripheral CNP | | | | | | | | | |
| Absent | 0 (0) | 0 (0) | 0 (0) | 1 (33.33) | 5 (4.59) | 0 (0) | 0 (0) | 0 (0) | 0 (0) |
| Grade 1 | 14 (93.33) | 2 (100) | 1 (100) | 2 (66.67) | 63 (57.8) | 0 (0) | 71 (47.33) | 1 (33.33) | 0 (0) |
| Grade 2 | 0 (0) | 0 (0) | 0 (0) | 0 (0) | 15 (13.76) | 1 (100) | 36 (24) | 1 (33.33) | 1 (100) |
| Grade 3 | 1 (6.67) | 0 (0) | 0 (0) | 0 (0) | 12 (11.01) | 0 (0) | 23 (15.33) | 0 (0) | 0 (0) |
| Grade 4 | 0 (0) | 0 (0) | 0 (0) | 0 (0) | 12 (11.01) | 0 (0) | 15 (10.00) | 0 (0) | 0 (0) |
| Cannot grade | 0 (0) | 0 (0) | 0 (0) | 0 (0) | 2 (1.83) | 0 (0) | 5 (3.33) | 1 (3.33) | 0 (0) |
| Punctate focal leak | | | | | | | | | |
| Absent | 14 (93.33) | 1 (50.00) | 1 (100) | 2 (66.67) | 78 (71.56) | 0 (0) | 109 (72.67) | 2 (66.67) | 1 (100) |
| Grade 1 | 1 (6.67) | 1 (50.00) | 0 (0) | 1 (33.33) | 23 (21.1) | 1 (100) | 32 (21.33) | 0 (0) | 0 (0) |
| Grade 2 | 0 (0) | 0 (0) | 0 (0) | 0 (0) | 4 (3.67) | 0 (0) | 5 (3.33) | 0 (0) | 0 (0) |
| Grade 3 | 0 (0) | 0 (0) | 0 (0) | 0 (0) | 3 (2.75) | 0 (0) | 1 (0.67) | 0 (0) | 0 (0) |
| Grade 4 | 0 (0) | 0 (0) | 0 (0) | 0 (0) | 0 (0) | 0 (0) | 1 (0.67) | 0 (0) | 0 (0) |
| Cannot grade | 0 (0) | 0 (0) | 0 (0) | 0 (0) | 1 (0.92) | 0 (0) | 2 (1.33) | 1 (3.33) | 0 (0) |
| Disc leak | | | | | | | | | |
| Absent | 11 (73.33) | 1 (50.00) | 0 (0) | 3 (100) | 24 (22.02) | 0 (0) | 18 (12.00) | 0 (0) | 1 (100) |
| Present | 4 (26.67) | 1 (50.00) | 1 (100) | 0 (0) | 84 (77.06) | 1 (100) | 131 (87.33) | 2 (66.67) | 0 (0) |
| Disc not seen | 0 (0) | 0 (0) | 0 (0) | 0 (0) | 1 (0.92) | 0 (0) | 1 (0.67) | 1 (33.33) | 0 (0) |
| Post-capillary venule leak | | | | | | | | | |
| Absent | 14 (93.33) | 0 (0) | 1 (100) | 3 (100) | 62 (56.88) | 0 (0) | 66 (44.00) | 0 (0) | 1 (100) |
| Grade 1 | 1 (6.67) | 1 (50.00) | 0 (0) | 0 (0) | 28 (25.69) | 0 (0) | 40 (26.67) | 1 (33.33) | 0 (0) |
| Grade 2 | 0 (0) | 1 (50.00) | 0 (0) | 0 (0) | 12 (11.01) | 0 (0) | 24 (16.00) | 0 (0) | 0 (0) |
| Grade 3 | 0 (0) | 0 (0) | 0 (0) | 0 (0) | 4 (3.67) | 1 (100) | 13 (8.67) | 1 (33.33) | 0 (0) |
| Grade 4 | 0 (0) | 0 (0) | 0 (0) | 0 (0) | 1 (0.92) | 0 (0) | 3 (2.00) | 0 (0) | 0 (0) |
| Cannot grade | 0 (0) | 0 (0) | 0 (0) | 0 (0) | 2 (1.83) | 0 (0) | 4 (2.67) | 1 (33.33) | 0 (0) |
| Large venule leak | | | | | | | | | |
| Absent | 12 (80.00) | 1 (50.00) | 1 (100) | 3 (100) | 79 (72.48) | 0 (0) | 80 (53.33) | 1 (33.33) | 1 (100) |
| Grade 1 | 3 (20.00) | 1 (50.00) | 0 (0) | 0 (0) | 22 (20.18) | 0 (0) | 50 (33.33) | 0 (0) | 0 (0) |
| Grade 2 | 0 (0) | 0 (0) | 0 (0) | 0 (0) | 5 (4.59) | 1 (100) | 12 (8.00) | 1 (33.33) | 0 (0) |
| Grade 3 | 0 (0) | 0 (0) | 0 (0) | 0 (0) | 1 (0.92) | 0 (0) | 6 (4.00) | 0 (0) | 0 (0) |
| Cannot grade | 0 (0) | 0 (0) | 0 (0) | 0 (0) | 2 (1.83) | 0 (0) | 2 (1.33) | 1 (33.33) | 0 (0) |
| IVFD, capillaries | | | | | | | | | |
| Absent | 7 (46.67) | 0 (0) | 0 (0) | 2 (66.67) | 31 (28.44) | 0 (0) | 30 (20.00) | 1 (33.33) | 0 (0) |
| Present | 0 (0) | 1 (50.00) | 0 (0) | 0 (0) | 18 (16.51) | 0 (0) | 20 (13.33) | 0 (0) | 0 (0) |
| Cannot grade | 8 (53.33) | 1 (50.00) | 1 (100) | 1 (33.33) | 60 (55.05) | 1 (100) | 100 (66.67) | 2 (66.67) | 1 (100) |
| IVFD, post-capillary venules | | | | | | | | | |
| Absent | 1 (6.67) | 1 (50.00) | 0 (0) | 1 (33.33) | 3 (2.75) | 0 (0) | 4 (2.67) | 0 (0) | 0 (0) |
| Present | 11 (73.33) | 1 (50.00) | 1 (100) | 2 (66.67) | 88 (80.73) | 0 (0) | 110 (73.33) | 2 (66.67) | 1 (100) |
| Cannot grade | 3 (20.00) | 0 (0) | 0 (0) | 0 (0) | 18 (16.51) | 1 (100) | 36 (24.00) | 1 (33.33) | 0 (0) |
| IVFD, pre-capillary arterioles | | | | | | | | | |
| Absent | 8 (53.33) | 2 (100) | 0 (0) | 2 (66.67) | 43 (39.45) | 0 (0) | 56 (37.33) | 2 (66.67) | 0 (0) |
| Present | 4 (26.67) | 0 (0) | 1 (100) | 1 (33.33) | 47 (43.12) | 0 (0) | 48 (32.00) | 0 (0) | 0 (0) |
| Cannot grade | 3 (20.00) | 0 (0) | 0 (0) | 0 (0) | 19 (17.43) | 1 (100) | 46 (30.67) | 1 (33.33) | 1 (100) |
| IVFD, small venules | | | | | | | | | |
| Absent | 5 (33.33) | 1 (50.00) | 0 (0) | 0 (0) | 4 (3.67) | 1 (100) | 13 (8.67) | 0 (0) | 0 (0) |
| Present | 9 (60.00) | 1 (50.00) | 1 (100) | 3 (100) | 98 (89.91) | 0 (0) | 126 (84.00) | 2 (66.67) | 1 (100) |
| Cannot grade | 1 (6.67) | 0 (0) | 0 (0) | 0 (0) | 7 (6.42) | 0 (0) | 11 (7.33) | 1 (33.33) | 0 (0) |
| IVFD, small arterioles | | | | | | | | | |
| Absent | 14 (93.33) | 2 (100) | 0 (0) | 1 (33.33) | 70 (64.22) | 1 (100) | 91 (60.67) | 2 (66.67) | 0 (0) |
| Present | 0 (0) | 0 (0) | 1 (100) | 2 (66.67) | 31 (28.44) | 0 (0) | 46 (30.67) | 0 (0) | 1 (100) |
| Cannot grade | 1 (6.67) | 0 (0) | 0 (0) | 0 (0) | 8 (7.34) | 0 (0) | 13 (8.67) | 1 (33.33) | 0 (0) |
| IVFD, large venules | | | | | | | | | |
| Absent | 12 (80.00) | 1 (50.00) | 0 (0) | 3 (100) | 23 (21.10) | 1 (100) | 46 (30.67) | 1 (33.33) | 0 (0) |
| Present | 3 (20.00) | 1 (50.00) | 1 (100) | 0 (0) | 79 (72.48) | 0 (0) | 94 (62.67) | 1 (33.33) | 1 (100) |
| Cannot grade | 0 (0) | 0 (0) | 0 (0) | 0 (0) | 7 (6.42) | 0 (0) | 10 (6.67) | 1 (33.33) | 0 (0) |
| IVFD, large arterioles | | | | | | | | | |
| Absent | 15 (100) | 1 (50.00) | 1 (100) | 3 (100) | 88 (80.73) | 1 (100) | 129 (86.00) | 2 (66.67) | 1 (100) |
| Present | 0 (0) | 1 (50.00) | 0 (0) | 0 (0) | 15 (13.76) | 0 (0) | 12 (8.00) | 0 (0) | 0 (0) |
| Cannot grade | 0 (0) | 0 (0) | 0 (0) | 0 (0) | 6 (5.5) | 0 (0) | 9 (6.00) | 1 (33.33) | 0 (0) |
| Large focal leak | | | | | | | | | |
| Absent | 15 (100) | 2 (100) | 1 (100) | 3 (100) | 99 (91.67) | 0 (0) | 127 (84.67) | 3 (100) | 0 (0) |
| Present | 0 (0) | 0 (0) | 0 (0) | 0 (0) | 9 (8.33) | 1 (100) | 23 (15.33) | 0 (0) | 1 (100) |
| Range | N/A | N/A | N/A | N/A | 0-30 | 12* | 0-32 | N/A | 7* |
| Cannot grade | 0 (0) | 0 (0) | 0 (0) | 0 (0) | 0 (0) | 0 (0) | 0 (0) | 0 (0) | 0 (0) |
| Data in this table is from the adjudicated grading for the left eye  Malarial retinopathy was assessed by bilateral dilated indirect ophthalmoscopy  *Only one subject with severe malarial anaemia, and one subject with a non-malarial diagnosis, had large focal leak. These numbers are absolute counts, not ranges.  CNP = Capillary non-perfusion  IVFD = Intravascular filling defects  N/A = Not applicable | | | | | | | | | |
